# The Impact of Introducing Malaria Rapid Diagnostic Tests on Fever Case Management: A Synthesis of Ten Studies from the ACT Consortium

**DOI:** 10.4269/ajtmh.16-0955

**Published:** 2017-08-07

**Authors:** Katia J. Bruxvoort, Baptiste Leurent, Clare I. R. Chandler, Evelyn K. Ansah, Frank Baiden, Anders Björkman, Helen E. D. Burchett, Siân E. Clarke, Bonnie Cundill, Debora D. DiLiberto, Kristina Elfving, Catherine Goodman, Kristian S. Hansen, S. Patrick Kachur, Sham Lal, David G. Lalloo, Toby Leslie, Pascal Magnussen, Lindsay Mangham-Jefferies, Andreas Mårtensson, Ismail Mayan, Anthony K. Mbonye, Mwinyi I. Msellem, Obinna E. Onwujekwe, Seth Owusu-Agyei, Mark W. Rowland, Delér Shakely, Sarah G. Staedke, Lasse S. Vestergaard, Jayne Webster, Christopher J. M. Whitty, Virginia L. Wiseman, Shunmay Yeung, David Schellenberg, Heidi Hopkins

**Affiliations:** 1 London School of Hygiene & Tropical Medicine, London, United Kingdom;; 2 Ghana Health Service, Accra, Ghana;; 3 Ensign College of Public Health, Kpong, Ghana;; 4 Karolinska Institutet, Stockholm, Sweden;; 5 Leeds Institute of Clinical Trials Research, University of Leeds, Leeds, United Kingdom;; 6 University of Gothenburg, Gothenburg, Sweden;; 7 University of Copenhagen, Copenhagen, Denmark;; 8 US Centers for Disease Control and Prevention, Atlanta, Georgia;; 9 Liverpool School of Tropical Medicine, Liverpool, United Kingdom;; 10 Centre for Medical Parasitology, University of Copenhagen and Copenhagen University Hospital, Copenhagen, Denmark;; 11 Department for Veterinary and Animal Sciences, University of Copenhagen, Copenhagen, Denmark;; 12 Uppsala University, Uppsala, Sweden;; 13 Health Protection Research Organisation, Kabul, Afghanistan;; 14 Ministry of Health, Kampala, Uganda;; 15 Makerere University School of Public Health, Kampala, Uganda;; 16 Zanzibar Malaria Elimination Programme, Tanzania;; 17 Department of Pharmacology and Therapeutics, University of Nigeria, Enugu, Nigeria;; 18 Kintampo Health Research Centre, Kintampo, Ghana;; 19 Centre for Malaria Research, Karolinska Institutet, Stockholm, Sweden;; 20 Health Metrics at Sahlgrenska Academy, University of Gothenburg, Gothenburg, Sweden;; 21 Department of Infectious Disease Epidemiology and Prevention, Statens Serum Institut, Copenhagen, Denmark;; 22 School of Public Health and Community Medicine, University of New South Wales, Sydney, Australia

## Abstract

Since 2010, the World Health Organization has been recommending that all suspected cases of malaria be confirmed with parasite-based diagnosis before treatment. These guidelines represent a paradigm shift away from presumptive antimalarial treatment of fever. Malaria rapid diagnostic tests (mRDTs) are central to implementing this policy, intended to target artemisinin-based combination therapies (ACT) to patients with confirmed malaria and to improve management of patients with nonmalarial fevers. The ACT Consortium conducted ten linked studies, eight in sub-Saharan Africa and two in Afghanistan, to evaluate the impact of mRDT introduction on case management across settings that vary in malaria endemicity and healthcare provider type. This synthesis includes 562,368 outpatient encounters (study size range 2,400–432,513). mRDTs were associated with significantly lower ACT prescription (range 8–69% versus 20–100%). Prescribing did not always adhere to malaria test results; in several settings, ACTs were prescribed to more than 30% of test-negative patients or to fewer than 80% of test-positive patients. Either an antimalarial or an antibiotic was prescribed for more than 75% of patients across most settings; lower antimalarial prescription for malaria test-negative patients was partly offset by higher antibiotic prescription. Symptomatic management with antipyretics alone was prescribed for fewer than 25% of patients across all scenarios. In community health worker and private retailer settings, mRDTs increased referral of patients to other providers. This synthesis provides an overview of shifts in case management that may be expected with mRDT introduction and highlights areas of focus to improve design and implementation of future case management programs.

## INTRODUCTION

Providing appropriate antimalarial treatment to patients who have malaria has been a long-standing challenge in fever case management and has traditionally relied on presumptive symptom-based diagnosis. Many people with malaria do not receive effective antimalarial medications, increasing their risk of severe disease or death. At the same time, many of those who receive antimalarials do not have malaria and are suffering from a nonmalaria illness which may need alternative treatment.^1^ To improve the rational use of artemisinin-based combination therapies (ACTs), the World Health Organization (WHO) recommended in 2010 that all suspected cases of malaria should have parasitological confirmation before treatment.^2,3^ These changes represent a paradigm shift from presumptive antimalarial treatment of fever to targeted use of ACTs only for those with a positive malaria test.

Central to implementing this policy change are malaria rapid diagnostic tests (mRDTs), relatively simple, inexpensive, and reliable point-of-care tests that can be used where high-quality microscopy services are not available.^4^ mRDTs are intended to improve the management of suspected malaria cases, increasing the use of first-line antimalarials in patients with confirmed malaria and encouraging the diagnosis and appropriate treatment of patients without malaria.^1^ After the WHO policy change, mRDT procurement surged from 45 million tests globally in 2008 to 314 million in 2014.^5^ Parasite-based diagnosis before treatment is now a policy in public health facilities in most malaria-endemic countries, and mRDTs are also being introduced among private retail and community health providers.^6–14^

Clinical trials and early pilot projects before the widespread adoption of mRDTs supported their use, though with some heterogeneity of results.^15^ Compared with presumptive treatment with antimalarials, case management based on mRDTs generally reduced antimalarial prescription, particularly in settings with relatively high provider adherence to test results and low malaria prevalence.^16–22^ On the other hand, although provider adherence to negative mRDT results was high in some studies,^16,17,23,24^ it was low in others.^25–27^ Comparable data from good-quality studies in a variety of contexts are needed to anticipate the effects of mRDT implementation as these tests are rolled out at scale.

The ACT Consortium is a research partnership created to address key questions and inform policy on ACT delivery.^28^ The Consortium conducted studies in 10 countries in Africa and Asia, including 10 studies specifically designed to address questions on improving the targeting of ACTs through the use of mRDTs. These studies looked at the impact of mRDT introduction on fever case management across a range of clinical and epidemiological contexts and among various types of healthcare providers. Studies evaluated different mRDT intervention packages, leading to heterogeneity that precludes formal meta-analysis. The current synthesis compares individual study results to identify patterns across contexts and provide an overview of what may be expected from mRDT implementation programs.

## METHODS

### Studies included in the analysis.

ACT Consortium studies were included in this analysis if they collected data on patient consultations for suspected malaria, evaluated an intervention to implement mRDTs by healthcare providers, and included a comparison group without the mRDT intervention. The 10 studies meeting these criteria are described in Table 1, including the abbreviation for each study used throughout the text. All studies received ethical approval from their host academic institutions and national authorities; see open-access publications for further details.^29–38^ Data are available at the ACT Consortium data repository (https://actc.lshtm.ac.uk/) or from the authors on request.

**Table 1:** Description of studies included in the analysis

Study country (reference)	Context	Healthcare provider type	Dates	Design	Setting*	Scenario description†	Number of patients	Number of clusters‡
Afgh1 Afghanistan (29)	Urban and rural	Public health facilities	September 2009–September 2010	Individually randomized trial	Afgh1/a	C	2,005	12
						R1	2,048	12, same as C
					Afgh1/b	C	517	5
						R1	527	5, same as C
					Afgh1/c	C	323	5
						R1	329	5, same as C
Afgh2 Afghanistan (30)	Urban and rural	Community health workers	October 2011–May 2012	Cluster-randomized trial	Afgh2/a	C	607	6
						R1	733	6
					Afgh2/b	C	594	5
						R1	466	5
Cam1 Cameroon (31)	Urban and rural	Public and mission health facilities	October–December 2011	Cluster-randomized trial	Cam1/a	C	400	5
						R1	699	8
						R2	778	9
					Cam1/b	C	281	4
						R1	932	10
						R2	891	10
Ghan1 Ghana (32)	Rural	Public health facilities	August 2007–December 2008	Individually randomized trial	Ghan1/a	C	1,907	1
						R1	1,904	1, same as C
					Ghan1/b	C	1,727	3
						R1	1,725	3, same as C
Nige1 Nigeria (33)	Urban and rural	Public health facilities and private medicine retailers	July–December 2009 (formative), June–December 2011 (trial)	Formative study followed by cluster-randomized trial	Nige1	C	1,642	100
						R1	1,588	41
						R2	1,850	47
						R3§	1,508	41
Tanz1 Tanzania (34)	Rural/periurban	Public health facilities	May–October 2010 (baseline), April–July 2012 (follow-up)	Descriptive before and after evaluation	Tanz1/a	C	689	39
						R1	750	60
					Tanz1/b	C	559	56
						R1	388	60
					Tanz1/c	C	498	44
						R1	572	57
Tanz2 Tanzania (35)	Rural	Public health facilities	September 2010– January 2011 (baseline), February 2011–Mar. 2012 (trial)	Baseline, followed by cluster-randomized trial	Tanz2	C	16,068	36
						R1	14,217	12
						R2	15,931	12
						R3||	13,973	12
Uga1 Uganda (36)	Rural	Public health facilities	April 2011–March 2013	Cluster-randomized trial	Uga1	C	210,758	10
						R1	221,755	10
Uga2 Uganda (37)	Rural	Community health workers	January–December 2011	Cluster-randomized trial	Uga2/a	C	2,444	32
						R1	1,207	32
					Uga2/b	C	10,625	31
						R1	7,872	30
Uga3 Uganda (38)	Rural	Private medicine retailers	January–December 2011	Cluster-randomized trial	Uga3	C	8,109	10
						R2	10,365	10

Further details of the studies are available from individual study publications.

* Some studies had multiple “settings,” defined as distinct geographical areas, malaria transmission zones, or different standard practices of malaria diagnosis. Where the study had only one setting, the study and setting abbreviations are the same.

† C = Without malaria rapid diagnostic test (mRDT) interventions; R1 = mRDT intervention with basic provider training; R2 = mRDT intervention with enhanced provider training; R3 = mRDT intervention with enhanced provider training and other activities.

‡ Clusters were health facilities in all studies, except Nige1 (health facilities and private medicine retailers), Uga2 (villages) and Uga3 (drug shops within a single administrative area, and drug shops in a neighboring administrative area if the distance between drug shops was < 1 km).

§ The R3 intervention in Nige1 also included school-based activities.

|| The R3 intervention in Tanz2 also included patient sensitization.

Eight studies took place in sub-Saharan Africa and two in Afghanistan, in a mix of rural and urban settings. mRDTs were introduced in health facilities only (Afgh1, Cam1, Ghan1, Tanz1, Tanz2, and Uga1), among community health workers (Afgh2 and Uga2), in private drug shops only (Uga3), or in a combination of public facilities, private pharmacies, and drug shops (Nige1). Seven studies were cluster-randomized trials of interventions to introduce mRDTs, two studies were individually randomized trials (Afgh1 and Ghan1), and one study was a descriptive “before and after” evaluation (Tanz1). All patients that were eligible in each study were included in the present analysis; typically, these were patients with suspected malaria, although one study included only children under age 5 years (Uga2), and two studies collected data on all patient consultations (Tanz2 and Uga1). Data were collected using provider-completed records of treatments administered (Afgh1, Afgh2, Ghan1, Uga1, and Uga2), patient exit interviews (Tanz1), both of these methods (Cam1, Nige1, and Tanz2), or provider-completed records with follow-up interviews of a subsample of patients (Uga3).

From each study, “settings” and “scenarios” were identified for this analysis. Six studies were conducted in multiple settings (indicated by suffix a, b, and c), such as distinct geographical areas and malaria transmission zones (Afgh1, Afgh2, Cam1, Tanz1, and Uga2), or where providers used different methods of routine malaria diagnosis (presumptive care or microscopy; Afgh1 and Ghan1). Trial arms or comparison groups within a setting were termed scenarios. All settings included at least one scenario without mRDT interventions, and settings in three studies (Cam1, Nige1, and Tanz2) included multiple mRDT intervention scenarios. In total, the 10 studies were conducted in 18 settings, with 18 scenarios without mRDT interventions and 24 scenarios with mRDT interventions.

Data were collected concurrently from scenarios with and without mRDT interventions in seven studies. In three studies (Nige1, Tanz1, and Tanz2), data from scenarios without mRDT interventions were collected before mRDT introduction. The scale of the interventions and their evaluations varied: for example, in Uga1 the intervention was implemented in 10 health facilities, and data were collected on 432,513 patient encounters in the study area whereas Tanz1 evaluated a nationwide intervention, and data were collected from 3,456 patients.

Microscopy was widely available in all settings in Cam1 and available at some higher-level facilities in Tanz1, particularly in the Tanz1/c scenario without mRDT interventions. The two individually randomized studies (Afgh1 and Ghan1) took place both in settings where microscopy was the standard practice and in settings where malaria diagnosis was symptom based. Microscopy services were nonexistent or very limited in the other six studies (Afgh2, Nige1, Tanz2, Uga1, Uga2, and Uga3).

### Indicators of interest.

To examine the impact of mRDTs on patient care, malaria testing and prescribing indicators were reviewed. Because the objective was to compare case management in areas with and without mRDT interventions, the first indicator of interest was the proportion of patients tested by the provider with any parasite-based diagnostic test (microscopy or mRDT). Prescribing indicators were the proportions of patients prescribed one or more of the following medicines: ACTs, non-ACT antimalarials, antibiotics (antibacterials), antifungals, antihelminthics, and antipyretics. The proportion of patients referred to another healthcare provider was also reviewed.

The ACT indicator was adjusted to account for malaria epidemiology and differences in first-line antimalarial in two cases: In Afghanistan, *Plasmodium vivax* was treated with chloroquine and *Plasmodium falciparum* with ACT; in these settings, the proportion of patients prescribed any antimalarial is reported instead of ACT. In Nige1, prescription of sulfadoxine-pyrimethamine (SP) and ACTs are reported for the scenario without mRDT interventions, whereas only ACTs are reported for the scenarios with mRDT interventions. This reflects a change in treatment between the 2009 scenario without mRDT interventions (when ACTs were recommended but not yet widely used) and the 2011 scenarios with mRDT interventions (when ACTs had largely replaced SP).

### Analytical approach.

Descriptive statistics on the indicators of interest were calculated from each scenario. Estimates for each indicator were made for scenarios without mRDT interventions and those with mRDT interventions. Prescribing indicators were further stratified by result of the diagnostic test performed by the healthcare provider. Odds ratios and 95% confidence intervals for indicators of interest within each setting were calculated using logistic regression with robust standard errors to account for clustering by the primary unit of sampling or randomization (see Supplemental Tables). Formal meta-analysis was deemed inappropriate because of the heterogeneity of interventions evaluated and study contexts. However, to aid comparisons between scenarios with and without mRDT interventions, the indicators of interest are presented as graphic point estimates by study arm. The analysis was conducted in STATA 14 (STATA Corp LP, College Station, TX). Factors which may explain variations in mRDT use are examined with additional qualitative data sources elsewhere.^39^

## RESULTS

### Proportion of patients tested.

**Figure 1.**
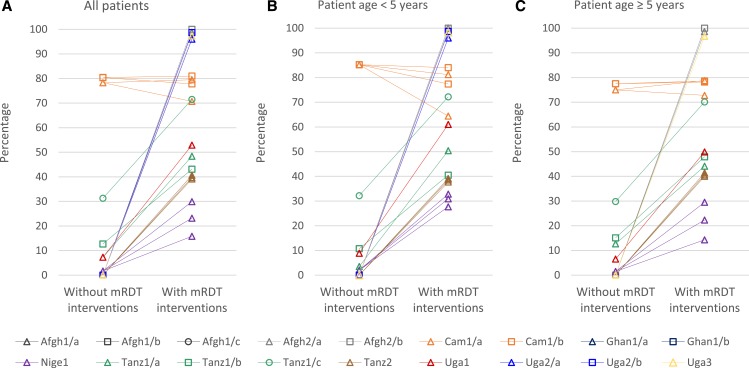
Patients in scenarios without and with malaria rapid diagnostic test (mRDT) interventions that were tested with any malaria diagnostic test at the provider of (**A**) all patients, (**B**) patients under age five years, and (**C**) patients ages five years and older. Afgh1 and Ghan1 studies individually randomized patients to malaria diagnostic method and are not included in this analysis. Some settings had more than one mRDT intervention scenario, which are graphed separately using the color and symbol for the setting. These include Cam1/a and Cam1/b (two intervention scenarios each), Nige1 (three intervention scenarios), and Tanz2 (three intervention scenarios). See Table 1. Scenarios with denominators fewer than 50 patients in Figure 2B are Afgh2/a without mRDT interventions and Afgh2/b both with and without mRDT interventions.

More patients were tested in scenarios where mRDTs had been introduced (Figure 1 and Supplemental Tables 1 3). However, even with mRDTs available, the percentage of patients tested varied widely, with 50% or fewer patients tested in five settings (Nige1, Tanz1/a, Tanz1/b, Tanz2, and Uga1), and nearly 100% in others (Afgh2/a, Afgh2/b, Uga/2, Uga2/b, and Uga3). The largest increases in proportion of patients tested were seen where mRDTs were introduced outside of health facilities (Afgh2, Uga2, and Uga3). Similar proportions of children and adults were tested in most scenarios, but in Nige1, Tanz1/a, and Uga1 test uptake was slightly higher for young children than for older patients. The proportion of patients tested is not reported in Afgh1 or Ghan1, where patients were individually randomized to mRDTs or microscopy (Afgh1/a, Afg1/b, and Ghan1/a), and to mRDTs or symptom-based diagnosis (Afgh1/c and Ghan1/b).

Patients were also tested with microscopy in Cam1 and, to a lesser extent, in Tanz1. In Cam1/a and Cam1/b, microscopy was common in all scenarios, and test use was not higher in scenarios with mRDT interventions. In scenarios without mRDT interventions, 80% of patients were tested with microscopy. In the four scenarios with mRDT interventions, 27–61% of patients were tested with microscopy and 17–52% with mRDT (71–81% tested overall). Of the three Tanz1 settings, microscopy was most frequently used in the Tanz1/c scenario without mRDT interventions, where 29% of patients were tested with microscopy and 2% with mRDT; in the corresponding scenario with mRDT interventions, 8% were tested with microscopy and 63% with mRDT.

### Prescription of ACTs and other antimalarial medications.

**Figure 2.**
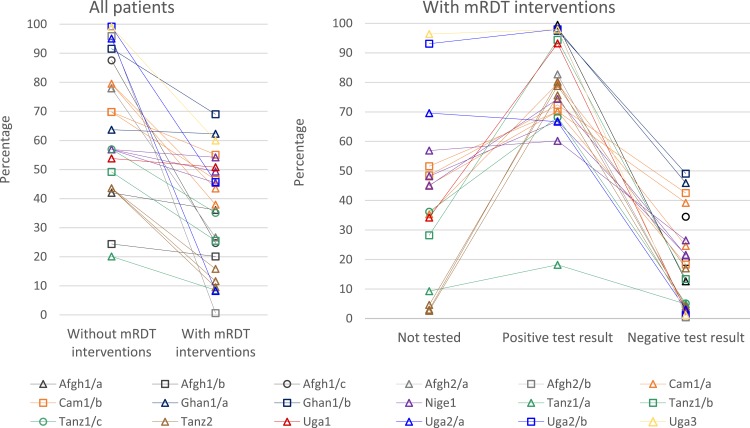
Patients prescribed an artemisinin-based combination therapy (ACT) of all patients in scenarios without and with malaria rapid diagnostic test (mRDT) interventions and by test result for all patients in scenarios with mRDT interventions. Graphs depict the percentage of patients prescribed ACT except for: Afgh1 and Afgh2, where all antimalarials are included to account for *Plasmodium vivax* treatment; and Nige1 without mRDT interventions only, where ACT or sulfadoxine-pyrimethamine (SP) are included to reflect treatment practices at the time of data collection. Scenarios with denominators fewer than 10 patients are not graphed, resulting in some points without adjoining lines: Afgh2/a and Afgh2/b in the “Not tested” column and Afgh1/b, Afgh1/c, and Afgh2/b in the “Positive test result” column. Afgh1 and Ghan1 studies individually randomized patients to malaria diagnostic method; data are not included in the “Not tested” column because all patients in mRDT intervention scenarios were tested. Some settings had more than one mRDT intervention scenario, which are graphed separately using the color and symbol for the setting. These include Cam1/a and Cam1/b (two intervention scenarios each), Nige1 (three intervention scenarios), and Tanz2 (three intervention scenarios). See Table 1. The following scenarios with denominators fewer than 50 patients are included: Uga2 in the “Not tested” column, and Cam1/a (R1), Tanz1/b, and Uga2/a in the “Positive test result” column. All other scenarios had larger denominators.

Overall, mRDTs were associated with lower ACT prescribing (Figure 2A and Supplemental Table 4). In 10/13 African settings, mRDT scenarios had statistically significantly lower ACT prescriptions than scenarios without mRDT interventions. In two African settings, there was little difference between mRDT and non-mRDT scenarios: Uga1, a high-transmission area where a high proportion of patients required ACTs even after testing and Ghan1/a, where all non-mRDT patients were randomized to testing with microscopy. In Nige1, where levels of testing were very low, presumptive diagnosis of malaria was common even where mRDTs were available. Prescription of ACT or SP in the scenario without mRDT interventions was similar to prescription of ACT in the three mRDT intervention scenarios (around 50%). In 4/5 Afghanistan settings, prescription of any antimalarial was much lower in scenarios with mRDT interventions than without; the exception was Afgh1/b, where (similar to Ghan1/a) all non-mRDT patients were randomized to testing with microscopy and where malaria transmission was low.

Recorded prescription of non-ACT antimalarials (e.g., SP, quinine, oral, and artemisinin monotherapies) was generally uncommon, except in Afghanistan. In 11/13 African settings, non-ACTs were prescribed for fewer than 10% of patients both with and without mRDT interventions (data not shown). Prescription of non-ACT antimalarials was higher in Cam1/b (20.9% in the scenario without an mRDT intervention and approximately 15% in the two scenarios with mRDT interventions) and in Nige1 (52.8% in the scenario without an mRDT intervention and approximately 30% in the three scenarios with mRDT interventions).

Overall, the finding of lower ACT prescription in scenarios with mRDT interventions was mostly due to malaria test-negative patients not receiving ACTs (Figure 2B–D and Supplemental Table 5). Fewer than 30% of test-negative patients were treated with ACTs in most mRDT intervention scenarios; exceptions were Cam1/a and Cam1/b, and Ghan1/a and Ghan1/b, where ACTs were prescribed for 39.2–49.1% of patients with negative malaria test results. There was no evident difference in this indicator by test type; in the Cam1/a and Cam1/b scenarios with mRDT interventions, ACTs were prescribed to 17.3–42.9% of microscopy test-negative patients and 15.6–45.9% of mRDT test-negative patients (data not shown). The percentages of malaria test-positive patients in scenarios with mRDT interventions who were prescribed ACTs ranged from 60.2% to 98.0% in 12/15 settings with data for this indicator. Prescription of ACTs to test-positive patients was over 90% in six of these settings, but was just 60.2–81.2% in another six settings, with 69.4–96.2% prescribed any antimalarial. In Tanz1/a, where stock-outs of ACTs in public health facilities were a major problem, ACT prescribing for test-positive patients was 18.2%. In Afgh1/a and Afgh2/a, 99.5% and 82.7% of test-positive patients were prescribed any antimalarial.

### Prescription of antibiotics.

**Figure 3.**
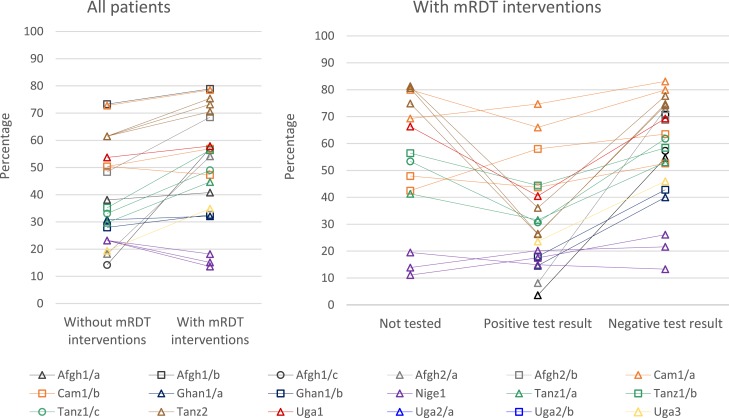
Patients prescribed an antibacterial of all patients in scenarios without and with malaria rapid diagnostic test (mRDT) interventions and by test result for all patients in scenarios with mRDT interventions. Some settings had more than one mRDT intervention scenario, which are graphed separately using the color and symbol for the setting. These include Cam1/a and Cam1/b (two intervention scenarios each), Nige1 (three intervention scenarios), and Tanz2 (three intervention scenarios). See Table 1. Community health workers in Uga2 were not permitted to prescribe antibacterials medications, so this study is not included in figure 3. Afgh1 and Ghan1 studies individually randomized patients to malaria diagnostic method; data are not included in the “Not tested” column because all patients in scenarios with mRDT interventions were tested. Scenarios with denominators fewer than 10 patients are not graphed, resulting in some points without adjoining lines: Afgh2/a and Afgh2/b in the “Not tested” column, and Afgh1/b, Afgh1/c, and Afgh2/b in the “Positive test result” column. The following scenarios with denominators fewer than 50 patients are included: Cam1/a (R1) and Tanz1/b in the “Positive test result” column. All other scenarios had larger denominators.

**Figure 4.**
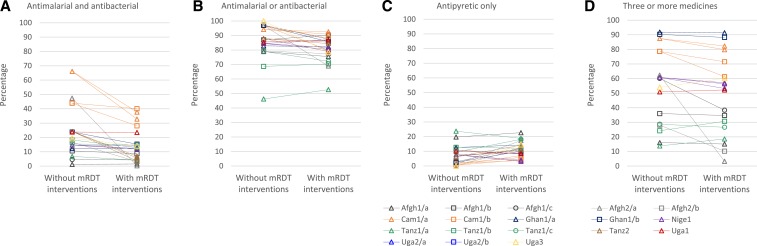
Patients in scenarios without and with malaria rapid diagnostic test (mRDT) interventions prescribed (**A**) an antimalarial and an antibacterial, (**B**) an antimalarial or an antibacterial, (**C**) an antipyretic without an antimalarial or an antibacterial, and (**D**) three or more medicines. Some settings had more than one mRDT intervention scenario, which are graphed separately using the color and symbol for the setting. These include Cam1/a and Cam1/b (two intervention scenarios each); Nige1 (three intervention scenarios) and Tanz2 (three intervention scenarios). See Table 1. Community health workers in Uga2 were not permitted to prescribe antibacterials medications, so this study is not included in figure 4. Tanz2 did not record data on all medications prescribed, so this study is not included in (**D**).

In contrast to reduced ACT prescribing, the mRDT interventions were associated with significantly more prescribing of systemic antibiotic (antibacterial) medications in seven settings (Afgh1/c, Afgh2/a, Tanz1/a, Tanz1/b, Tanz1/c, Tanz2, and Uga3) (Figure 3 and Supplemental Tables 6 7). In scenarios with mRDT interventions, antibiotic prescribing patterns varied by mRDT result. In all settings except Nige1, 40.0–79.9% of patients who tested negative for malaria were prescribed antibiotics. Antibiotic prescription was similar in patients who were not tested. Among those with a positive malaria test result, fewer than 45% were prescribed antibiotics, with higher proportions in Cam1/a and Cam1/b. Prescription of both an antimalarial and a systemic antibiotic (Figure 4A and Supplemental Table 8) was relatively uncommon in all settings (< 25% of patients, except in Cam1 and Afgh2/b) and was similar or lower in scenarios with mRDT interventions. By contrast, the prescription of either an antimalarial or an antibiotic medicine was high in all settings (more than 68%, except in Tanz1/a) and similar or lower in scenarios with mRDT interventions (Figure 4B and Supplemental Table 9). Further details of antibiotic prescribing in ACT Consortium studies are presented elsewhere.^40^

### Prescription of other medicines.

Data were recorded on prescription of other anti-infectives in some study settings. Prescription of systemic antifungals (fluconazole and griseofulvin) was reported in five settings (Cam1/a, Cam1/b, Ghan1/a, Ghan1/b, and Uga1); the proportion of patients prescribed these medicines across these settings was 2.6% or less (Supplemental Table 10). Prescription of antihelminthics (albendazole and mebendazole) was recorded in 13 settings (all study settings except those in Afgh2, Tanz2, and Uga2); the proportion of patients prescribed these medicines ranged from 0.3% to 33.3%, which did not appear attributable to whether the scenarios had an mRDT intervention or not (Supplemental Table 10).

Prescription of antipyretic medicines alone, for symptomatic relief, without an antimalarial or an antibiotic, ranged from 0.3% to 23.7% across all scenarios and was similar or higher with mRDT interventions except in Nige1 (Figure 4C and Supplemental Table 11). Polypharmacy, defined as the prescription of three or more medicines, varied widely across settings (Figure 4D). However, in most settings, polypharmacy was comparable with and without mRDT interventions, but was significantly lower with mRDT interventions in four settings (Afgh1/b, Afgh2/a, Afgh2/b, and Cam2/b (Figure 4D and Supplemental Table 12).

### Referral.

**Figure 5.**
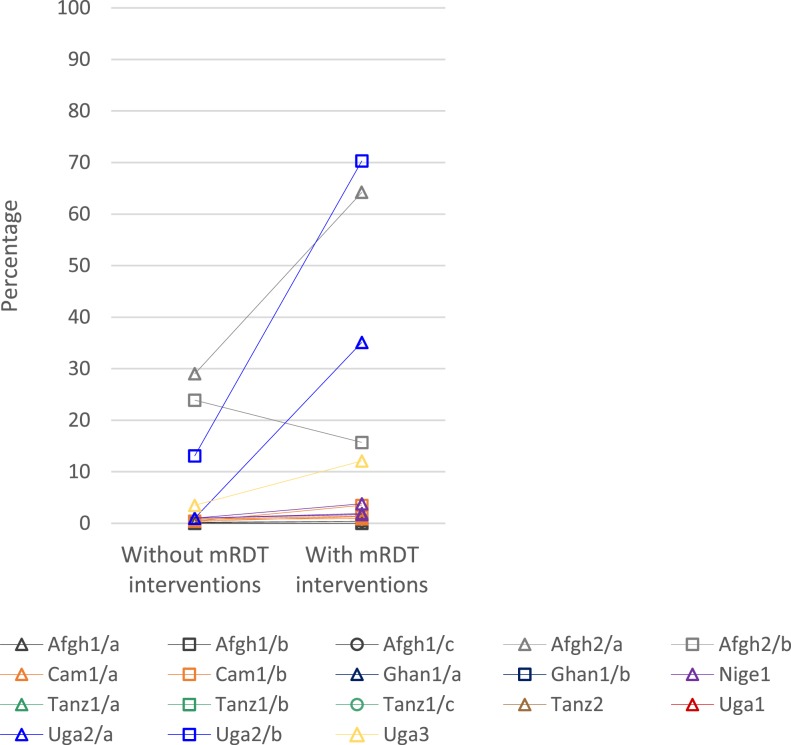
Patients in scenarios without and with malaria rapid diagnostic test (mRDT) interventions that were referred to another care provider or health facility. Ghan1, Tanz1, Tanz2, and Uga1 did not record data on referral. Case management was performed by community health workers in Afgh2 and Uga2, private drug store retailers in Uga3, and both public and private health facilities in Nige1. All other studies were conducted in public health facilities.

Figure 5 and Supplemental Table 13 show the percentage of patients referred to another care provider or facility. Referral was generally low across study settings. However, referral was significantly higher with mRDT interventions among community health workers, particularly in Uga2/a, Uga2/b, and Afgh2/b, and to a lesser extent in Uga3. Referral was uncommon (< 5%) across all scenarios in studies in public health facilities.

## DISCUSSION

Providing appropriate treatment to patients who present with malaria-like symptoms remains a challenge in many endemic regions. This synthesis of data from 10 ACT Consortium studies illustrates the impact of mRDTs on case management. The data represent 24 scenarios where mRDTs were introduced, compared with 18 scenarios without mRDT interventions. This synthesis found that mRDT interventions reduced prescription of first-line antimalarials across almost all settings, except where the tests were not often used. However, prescribing did not always reflect test results: across a range of scenarios, ACTs were prescribed for some mRDT-negative cases, and at least as concerning, ACTs were not prescribed for all mRDT-positive cases. The use of mRDTs also influenced other treatment decisions, notably resulting in an increase of antibiotic prescription especially for test-negative cases. Referral of patients to other healthcare providers was low across nearly all settings, with a few specific exceptions discussed below.

What lessons can be learned from this synthesis, to inform expectations of programs that implement mRDTs at scale? Although mRDTs generally improve malaria case management, alone, they are not a panacea to solve the major challenge of effective fever management. Simply providing mRDTs is insufficient if health workers continue prescribing antimalarials to test-negative patients^27,41^ or if alternative treatments are not appropriate. The ACT Consortium studies evaluated a range of tailored and pretested elements as part of mRDT intervention strategies, such as enhanced provider training or community awareness activities.^42,43^ Anecdotally, interventions designed with more intensive formative research led to greater reductions in ACT prescription for test-negative patients; but such prescribing remained inappropriately high (10–49%).

Furthermore, in five of the eight African studies included in this analysis, more than 20% of patients who tested positive for malaria at the point of care were not prescribed ACTs. Undertreatment of malaria in settings where mRDTs have been implemented has been recognized in a small proportion of cases (less than 5%), with few exceptions.^44–46^ However, results of this synthesis suggest that undertreatment may be a more common problem than previously recognized. The six settings with high ACT prescription for test-positive patients varied in terms of malaria epidemiology, geography, and provider type; the same is true for the six settings with lower ACT prescription for test-positive patients. To date, research into the reasons for this phenomenon has been limited, although ACT Consortium study results presented elsewhere suggest that provider motivations, stability of ACT supplies and preexisting antimalarial preferences account for some of this underprescription.^39^ Missed or ineffective treatment of malaria presents a risk to patients; a balance between reducing unnecessary antimalarial use while ensuring ACTs are provided to all malaria-positive cases needs to be integrated in future research, training, and implementation programs.

This synthesis highlights the fact that effecting change in one healthcare practice can have knock-on consequences for other practices. In many ACT Consortium studies, mRDT implementation was associated with a higher level of antibiotic prescription, particularly for malaria test-negative patients.^40^ The proportion of patients prescribed either an antimalarial or an antibiotic was high, for more than 75% of cases across most settings, and this was approximately similar in settings with and without mRDT interventions. This suggests that in the absence of other diagnostic options, presumptive antimalarial treatment may be exchanged for presumptive antibiotic treatment when mRDTs are introduced. Many patients with uncomplicated febrile illness are likely to improve with symptomatic management only (e.g., antipyretic), as noted in WHO case management guidelines^47,48^; this approach was prescribed for just 0–24% of patients in ACT Consortium studies. Inappropriate use of antimicrobials is of increasing global concern because of rising resistance, which can result in longer illnesses, higher mortality, and increased treatment costs.^49,50^ A more comprehensive approach to case management is needed, rather than focusing on only a single diagnosis and medication (e.g., malaria mRDTs and ACTs), if unintended consequences are to be avoided.^51^

Our data support the observation that introducing mRDTs may increase patient referral to other healthcare providers, particularly among community health workers and private retailers.^10,52^ In particular, when a malaria test is negative, alternative diagnoses must be considered; the clinical skills and diagnostic capacity to achieve this are limited among providers with less formal training, so that, referral may be necessary for adequate case management. Overall, referral remained infrequent in ACT Consortium studies. Even when referral is recommended, patients are not always inclined or able to follow the recommendation.^53–55^ If current recommendations to scale up mRDTs in community and private healthcare settings are implemented, to improve referral practices in a way that is safe for individual patients, and without unduly burdening other parts of the healthcare system, the role of mRDTs will need to be better integrated into local pathways of treatment seeking and care provision.^56–58^

The observed shifts in case management practices have cost implications for health systems and for patients. When mRDTs lead to reductions in ACT use, there can be substantial savings in ACT costs. However, additional costs are incurred for mRDT implementation: the tests themselves, alternative treatments provided to mRDT-negative patients, additional referrals, and the activities required for mRDT introduction, such as training, supervision, communication campaigns, and quality control. The overall cost impact in a given context will depend on several parameters, including the relative cost of ACTs and mRDTs, the amount of subsidy for each, the proportion of patients tested, the proportion who test positive, and provider adherence to test results. Analyses of the incremental economic cost per fever case managed have been published for four studies included in this synthesis. Where mRDTs were compared with microscopy (Afgh1, Ghan1, and Cam1), mRDTs were cost saving or costs were similar in Afghanistan,^59^ with an incremental provider cost per fever case managed ranging from 0.20 to 1.11 USD in in Ghana^60^ and Cameroon^61^ (2011 USD). Where mRDTs were compared with clinical diagnosis, the incremental provider cost per fever case managed ranged from 0.24 to 10.9 USD across different transmission levels and provider types in Afghanistan, Ghana, and Uganda (2011 USD).^59,60,62^ These incremental costs may be considered good value for money if they lead to sufficient improvements in health outcomes. A full consideration of cost effectiveness would require costs from both health sector and household perspectives, extrapolation to final health outcomes such as cost per death or disability adjusted life year averted, and sensitivity analyses to explore the impact of variation in prescribing and referral practices. Ideally, a full analysis should also include the impact of malaria testing on enhancing malaria surveillance systems and resulting improvements in targeting of malaria interventions.

The present analysis was subject to several limitations. Data were collected concurrently from scenarios with and without mRDT interventions in seven studies, whereas in the other three (Nige1, Tanz1, and Tanz2) data were collected before and after mRDT introduction (Table 1). In Nige1, the interval between the two data collection points corresponded with a shift in antimalarial use from SP to ACT; whereas ACT prescription decreased, any antimalarial prescription remained high (≥ 75%). In addition, some indicators varied in availability and precise definition across studies (see footnotes to Figures and Supplemental Tables). For example, in Uga2, prescription of antibiotics and polypharmacy was not reported because community health workers were only permitted to dispense antimalarials and antipyretics. In Tanz1, data on medicines prescribed were not available from scenarios without mRDTs, so data on medicines dispensed were used for all Tanz1 scenarios. In designing the ACT Consortium studies and mRDT implementation packages, investigators sought to accommodate varied and transitioning contexts while still obtaining data that could be compared across studies. This synthesis therefore did not aim to provide combined estimates of the size of effect of the impact of mRDTs (meta-analysis). Instead, comparison of findings from the individual studies identified clear patterns across diverse geographical, epidemiological, and health sector contexts, indicating both robustness and generalizability of the results.

In summary, evidence from ten ACT Consortium studies demonstrates that mRDT introduction can reduce prescription of ACTs. However, mRDTs are not an easy technological fix. Critically, challenges exist in ensuring that all patients who test positive for falciparum malaria are prescribed ACT; anything less endangers individual patients and the credibility of programs. It is also necessary to ensure that patients who test negative receive appropriate management, which may or may not include other antimicrobials. ACT Consortium studies were conducted between 2007 and 2013, and since that time, mRDT implementation programs continue to evolve. These combined results provide an overview of the generally positive shifts in case management that may be expected with mRDT introduction, and highlight issues that warrant particular attention in future work on point-of-care diagnosis and fever and malaria case management.

## Supplementary Material

Supplemental Tables.

## References

[1] Whitty CJ Chandler C Ansah E Leslie T Staedke SG, 2008 Deployment of ACT antimalarials for treatment of malaria: challenges and opportunities. Malar J 7 (Suppl 1): S7. 19091041 10.1186/1475-2875-7-S1-S7 PMC2604871

[2] World Health Organization, 2011 *Universal Access to Malaria Diagnostic Testing: An Operational Manual.* Geneva, Switzerland: World Health Organization.

[3] World Health Organization, 2010 *Guidelines for the Treatment of Malaria.* 2nd edition. Geneva, Switzerland: World Health Organization.

[4] Mabey D Peeling RW Ustianowski A Perkins MD, 2004 Diagnostics for the developing world. Nat Rev Microbiol 2: 231–240. 15083158 10.1038/nrmicro841

[5] UNITAID, 2016 *Malaria Diagnostics Landscape Update.* Geneva, Switzerland: World Health Organization for the UNITAID Secretariat.

[6] Ruizendaal E Dierickx S Peeters Grietens K Schallig HD Pagnoni F Mens PF, 2014 Success or failure of critical steps in community case management of malaria with rapid diagnostic tests: a systematic review. Malar J 13: 229. 24924295 10.1186/1475-2875-13-229 PMC4084582

[7] Mukanga D, 2012 Integrated community case management of fever in children under five using rapid diagnostic tests and respiratory rate counting: a multi-country cluster randomized trial. Am J Trop Med Hyg 87: 21–29. 23136274 10.4269/ajtmh.2012.11-0816 PMC3748518

[8] Hamer DH Brooks ET Semrau K Pilingana P MacLeod WB Siazeele K Sabin LL Thea DM Yeboah-Antwi K, 2012 Quality and safety of integrated community case management of malaria using rapid diagnostic tests and pneumonia by community health workers. Pathog Glob Health 106: 32–39. 22595272 10.1179/1364859411Y.0000000042 PMC4001509

[9] Awor P Wamani H Tylleskar T Jagoe G Peterson S, 2014 Increased access to care and appropriateness of treatment at private sector drug shops with integrated management of malaria, pneumonia and diarrhoea: a quasi-experimental study in Uganda. PLoS One 9: e115440. 25541703 10.1371/journal.pone.0115440 PMC4277343

[10] Ansah EK Narh-Bana S Affran-Bonful H Bart-Plange C Cundill B Gyapong M Whitty CJ, 2015 The impact of providing rapid diagnostic malaria tests on fever management in the private retail sector in Ghana: a cluster randomized trial. BMJ 350: h1019. 25739769 10.1136/bmj.h1019 PMC4353311

[11] Aung T White C Montagu D McFarland W Hlaing T Khin HS San AK Briegleb C Chen I Sudhinaraset M, 2015 Improving uptake and use of malaria rapid diagnostic tests in the context of artemisinin drug resistance containment in eastern Myanmar: an evaluation of incentive schemes among informal private healthcare providers. Malar J 14: 105. 25885581 10.1186/s12936-015-0621-7 PMC4355503

[12] Cohen J Fink G Maloney K Berg K Jordan M Svoronos T Aber F Dickens W, 2015 Introducing rapid diagnostic tests for malaria to drug shops in Uganda: a cluster-randomized controlled trial. Bull World Health Organ 93: 142–151.

[13] Yeung S Patouillard E Allen H Socheat D, 2011 Socially-marketed rapid diagnostic tests and ACT in the private sector: ten years of experience in Cambodia. Malar J 10: 243. 21851625 10.1186/1475-2875-10-243 PMC3173399

[14] UNITAID, 2016 *Creating a Private Sector Market for Quality-Assured RDTs in Malaria Endemic Countries.* Available at: http://www.unitaid.org/en/creating-a-private-sector-market-for-quality-assured-rdts-in-malaria-endemic-countries. Accessed October 21, 2016.

[15] Odaga J Sinclair D Lokong JA Donegan S Hopkins H Garner P, 2014 Rapid diagnostic tests versus clinical diagnosis for managing people with fever in malaria endemic settings. Cochrane Database Syst Rev 4: CD008998. 10.1002/14651858.CD008998.pub2 PMC4468923 24740584

[16] Msellem MI, 2009 Influence of rapid malaria diagnostic tests on treatment and health outcome in fever patients, Zanzibar: a crossover validation study. PLoS Med 6: e1000070. 19399156 10.1371/journal.pmed.1000070 PMC2667629

[17] Mubi M, 2011 Malaria rapid testing by community health workers is effective and safe for targeting malaria treatment: randomised cross-over trial in Tanzania. PLoS One 6: e19753. 21750697 10.1371/journal.pone.0019753 PMC3130036

[18] D’Acremont V Kahama-Maro J Swai N Mtasiwa D Genton B Lengeler C, 2011 Reduction of anti-malarial consumption after rapid diagnostic tests implementation in Dar es Salaam: a before-after and cluster randomized controlled study. Malar J 10: 107. 21529365 10.1186/1475-2875-10-107 PMC3108934

[19] Kyabayinze DJ Asiimwe C Nakanjako D Nabakooza J Counihan H Tibenderana JK, 2010 Use of RDTs to improve malaria diagnosis and fever case management at primary health care facilities in Uganda. Malar J 9: 200. 20624312 10.1186/1475-2875-9-200 PMC2914063

[20] Bastiaens GJ Schaftenaar E Ndaro A Keuter M Bousema T Shekalaghe SA, 2011 Malaria diagnostic testing and treatment practices in three different *Plasmodium falciparum* transmission settings in Tanzania: before and after a government policy change. Malar J 10: 76. 21457570 10.1186/1475-2875-10-76 PMC3080800

[21] Thiam S, 2011 Major reduction in anti-malarial drug consumption in Senegal after nation-wide introduction of malaria rapid diagnostic tests. PLoS One 6: e18419. 21494674 10.1371/journal.pone.0018419 PMC3071817

[22] Yukich JO, 2012 Reductions in artemisinin-based combination therapy consumption after the nationwide scale up of routine malaria rapid diagnostic testing in Zambia. Am J Trop Med Hyg 87: 437–446. 22848096 10.4269/ajtmh.2012.12-0127 PMC3435345

[23] Yeboah-Antwi K, 2010 Community case management of fever due to malaria and pneumonia in children under five in Zambia: a cluster randomized controlled trial. PLoS Med 7: e1000340. 20877714 10.1371/journal.pmed.1000340 PMC2943441

[24] Skarbinski J, 2009 Effect of malaria rapid diagnostic tests on the management of uncomplicated malaria with artemether-lumefantrine in Kenya: a cluster randomized trial. Am J Trop Med Hyg 80: 919–926. 19478249

[25] Bisoffi Z Sirima BS Angheben A Lodesani C Gobbi F Tinto H Van den Ende J, 2009 Rapid malaria diagnostic tests vs. clinical management of malaria in rural Burkina Faso: safety and effect on clinical decisions. A randomized trial. Trop Med Int Health 14: 491–498. 19222821 10.1111/j.1365-3156.2009.02246.x

[26] Chinkhumba J Skarbinski J Chilima B Campbell C Ewing V San Joaquin M Sande J Ali D Mathanga D, 2010 Comparative field performance and adherence to test results of four malaria rapid diagnostic tests among febrile patients more than five years of age in Blantyre, Malawi. Malar J 9: 209. 20646312 10.1186/1475-2875-9-209 PMC2916916

[27] Reyburn H Mbakilwa H Mwangi R Mwerinde O Olomi R Drakeley C Whitty CJ, 2007 Rapid diagnostic tests compared with malaria microscopy for guiding outpatient treatment of febrile illness in Tanzania: randomised trial. BMJ 334: 403. 17259188 10.1136/bmj.39073.496829.AE PMC1804187

[28] ACT Consortium, 2016 *ACT Consortium: Answering Key Questions on Malaria Drug Delivery.* Available at: http://www.actconsortium.org.

[29] Leslie T, 2014 Rapid diagnostic tests to improve treatment of malaria and other febrile illnesses: patient randomised effectiveness trial in primary care clinics in Afghanistan. BMJ 348: g3730. 24948695 10.1136/bmj.g3730 PMC4064827

[30] Leslie T, 2017 Use of malaria rapid diagnostic tests by community health workers in Afghanistan: cluster randomised trial. BMC Med. 15: 124. 28683750 10.1186/s12916-017-0891-8 PMC5501368

[31] Mbacham WF, 2014 Basic or enhanced clinician training to improve adherence to malaria treatment guidelines: a cluster-randomised trial in two areas of Cameroon. Lancet Glob Health 2: e346–e358. 25103303 10.1016/S2214-109X(14)70201-3

[32] Ansah EK Narh-Bana S Epokor M Akanpigbiam S Quartey AA Gyapong J Whitty CJ, 2010 Rapid testing for malaria in settings where microscopy is available and peripheral clinics where only presumptive treatment is available: a randomised controlled trial in Ghana. BMJ 340: c930. 20207689 10.1136/bmj.c930 PMC2833239

[33] Onwujekwe O Mangham-Jefferies L Cundill B Alexander N Langham J Ibe O Uzochukwu B Wiseman V, 2015 Effectiveness of provider and community interventions to improve treatment of uncomplicated malaria in Nigeria: a cluster randomized controlled trial. PLoS One 10: e0133832. 26309023 10.1371/journal.pone.0133832 PMC4550271

[34] Bruxvoort K, 2013 Getting antimalarials on target: impact of national roll-out of malaria rapid diagnostic tests on health facility treatment in three regions of Tanzania. Trop Med Int Health 18: 1269–1282. 23937722 10.1111/tmi.12168 PMC4282336

[35] Cundill B, 2015 Prescriber and patient-oriented behavioural interventions to improve use of malaria rapid diagnostic tests in Tanzania: facility-based cluster randomised trial. BMC Med 13: 118. 25980737 10.1186/s12916-015-0346-z PMC4445498

[36] Staedke SG, 2016 The impact of an intervention to improve malaria care in public health centers on health indicators of children in Tororo, Uganda (PRIME): a cluster-randomized trial. Am J Trop Med Hyg 95: 358–367. 27273646 10.4269/ajtmh.16-0103 PMC4973182

[37] Ndyomugyenyi R Magnussen P Lal S Hansen K Clarke SE, 2016 Appropriate targeting of artemisinin-based combination therapy by community health workers using malaria rapid diagnostic tests: findings from randomized trials in two contrasting areas of high and low malaria transmission in south-western Uganda. Trop Med Int Health 21: 1157–1170. 27383558 10.1111/tmi.12748 PMC5031222

[38] Mbonye AK Magnussen P Lal S Hansen KS Cundill B Chandler C Clarke SE, 2015 A cluster randomised trial introducing rapid diagnostic tests into registered drug shops in Uganda: impact on appropriate treatment of malaria. PLoS One 10: e0129545. 26200467 10.1371/journal.pone.0129545 PMC4511673

[39] Burchett HE, 2017 Improving prescribing practices with rapid diagnostic tests (RDTs): synthesis of 10 studies to explore reasons for variation in malaria RDT uptake and adherence. BMJ Open 7: e012973. 10.1136/bmjopen-2016-012973 PMC5353269 28274962

[40] Hopkins H, 2017 Impact of introduction of rapid diagnostic tests for malaria on antibiotic prescribing: analysis of observational and randomised studies in public and private healthcare settings. BMJ 356: j1054. 28356302 10.1136/bmj.j1054 PMC5370398

[41] Hamer DH Ndhlovu M Zurovac D Fox M Yeboah-Antwi K Chanda P Sipilinyambe N Simon JL Snow RW, 2007 Improved diagnostic testing and malaria treatment practices in Zambia. JAMA 297: 2227–2231. 17519412 10.1001/jama.297.20.2227 PMC2674546

[42] Achonduh OA, 2014 Designing and implementing interventions to change clinicians’ practice in the management of uncomplicated malaria: lessons from Cameroon. Malar J 13: 204. 24885621 10.1186/1475-2875-13-204 PMC4041055

[43] Chandler CI Meta J Ponzo C Nasuwa F Kessy J Mbakilwa H Haaland A Reyburn H, 2014 The development of effective behaviour change interventions to support the use of malaria rapid diagnostic tests by Tanzanian clinicians. Implement Sci 9: 83. 24969367 10.1186/1748-5908-9-83 PMC4227094

[44] Rao VB Schellenberg D Ghani AC, 2013 Overcoming health systems barriers to successful malaria treatment. Trends Parasitol 29: 164–180. 23415933 10.1016/j.pt.2013.01.005

[45] Masanja IM Selemani M Amuri B Kajungu D Khatib R Kachur SP Skarbinski J, 2012 Increased use of malaria rapid diagnostic tests improves targeting of anti-malarial treatment in rural Tanzania: implications for nationwide rollout of malaria rapid diagnostic tests. Malar J 11: 221. 22747655 10.1186/1475-2875-11-221 PMC3471012

[46] Nicastri E, 2009 Accuracy of malaria diagnosis by microscopy, rapid diagnostic test, and PCR methods and evidence of antimalarial overprescription in non-severe febrile patients in two Tanzanian hospitals. Am J Trop Med Hyg 80: 712–717. 19407111

[47] World Health Organization, 2011 *IMAI District Clinician Manual: Hospital Care for Adolescents and Adults, Guidelines for the Management of Common Illnesses with Limited Resources*. Integrated Management of Adolescent and Adult Illness (IMAI). Geneva, Switzerland: WHO.

[48] World Health Organization, 2014 *Integrated Management of Childhood Illness: Chart Booklet*. Geneva, Switzerland: WHO.

[49] Bebell LM Muiru AN, 2014 Antibiotic use and emerging resistance: how can resource-limited countries turn the tide? Glob Heart 9: 347–358. 25667187 10.1016/j.gheart.2014.08.009 PMC4369554

[50] Laxminarayan R, 2013 Antibiotic resistance-the need for global solutions. Lancet Infect Dis 13: 1057–1098. 24252483 10.1016/S1473-3099(13)70318-9

[51] Rambaud-Althaus C Shao AF Kahama-Maro J Genton B d’Acremont V, 2015 Managing the sick child in the era of declining malaria transmission: development of ALMANACH, an electronic algorithm for appropriate use of antimicrobials. PLoS One 10: e0127674. 26161753 10.1371/journal.pone.0127674 PMC4498609

[52] Lal S Ndyomugenyi R Magnussen P Hansen KS Alexander ND Paintain L Chandramohan D Clarke SE, 2016 Referral patterns of community health workers diagnosing and treating malaria: cluster-randomized trials in two areas of high- and low-malaria transmission in southwestern Uganda. Am J Trop Med Hyg 95: 1398–1408. 27799650 10.4269/ajtmh.16-0598 PMC5154457

[53] Newbrander W Ickx P Werner R Mujadidi F, 2012 Compliance with referral of sick children: a survey in five districts of Afghanistan. BMC Pediatr 12: 46. 22540424 10.1186/1471-2431-12-46 PMC3422210

[54] Thomson A Khogali M de Smet M Reid T Mukhtar A Peterson S von Schreeb J, 2011 Low referral completion of rapid diagnostic test-negative patients in community-based treatment of malaria in Sierra Leone. Malar J 10: 94. 21496333 10.1186/1475-2875-10-94 PMC3102648

[55] Kallander K Tomson G Nsungwa-Sabiiti J Senyonjo Y Pariyo G Peterson S, 2006 Community referral in home management of malaria in western Uganda: a case series study. BMC Int Health Hum Rights 6: 2. 16539744 10.1186/1472-698X-6-2 PMC1434779

[56] Obrist B, 2007 Access to health care in contexts of livelihood insecurity: a framework for analysis and action. PLoS Med 4: 1584–1588. 17958467 10.1371/journal.pmed.0040308 PMC2039761

[57] Altaras R Nuwa A Agaba B Streat E Tibenderana JK Martin S Strachan CE, 2016 How do patients and health workers interact around malaria rapid diagnostic testing, and how are the tests experienced by patients in practice? A qualitative study in western Uganda. PLoS One 11: e0159525. 27494507 10.1371/journal.pone.0159525 PMC4975385

[58] Peterson S Nsungwa-Sabiiti J Were W Nsabagasani X Magumba G Nambooze J Mukasa G, 2004 Coping with paediatric referral–Ugandan parents’ experience. Lancet 363: 1955–1956. 15194257 10.1016/S0140-6736(04)16411-8

[59] Hansen KS, 2015 Cost-effectiveness of malaria diagnosis using rapid diagnostic tests compared to microscopy or clinical symptoms alone in Afghanistan. Malar J 14: 217. 26016871 10.1186/s12936-015-0696-1 PMC4450447

[60] Ansah EK Epokor M Whitty CJ Yeung S Hansen KS, 2013 Cost-effectiveness analysis of introducing RDTs for malaria diagnosis as compared to microscopy and presumptive diagnosis in central and peripheral public health facilities in Ghana. Am J Trop Med Hyg 89: 724–736. 23980131 10.4269/ajtmh.13-0033 PMC3795104

[61] Mangham-Jefferies L Wiseman V Achonduh OA Drake TL Cundill B Onwujekwe O Mbacham W, 2014 Economic evaluation of a cluster randomized trial of interventions to improve health workers’ practice in diagnosing and treating uncomplicated malaria in Cameroon. Value Health 17: 783–791. 25498773 10.1016/j.jval.2014.07.010

[62] Hansen KS Ndyomugyenyi R Magnussen P Lal S Clarke SE, 2017 Cost-effectiveness analysis of malaria rapid diagnostic tests for appropriate treatment of malaria at the community level in Uganda. Health Policy Plan 32: 676–689. 28453718 10.1093/heapol/czw171 PMC5406761

