# Supplementary material for: The Impact of Introducing Malaria Rapid Diagnostic Tests on Fever Case Management: A Synthesis of Ten Studies from the ACT Consortium
Source: Am J Trop Med Hyg. 2017 Aug 7;97(4):1170–9. doi: 10.4269/ajtmh.16-0955 (PMC5637593; doi:10.4269/ajtmh.16-0955)
Supplement: Supplementary file 1 [file tpmd160955.SD1.pdf]

Supplemental Table 1

Patients tested by any malaria diagnostic test

Study (by setting)	Without mRDT interventions percentage numerator/ denominator	With mRDT interventions percentage numerator/ denominator	Odds ratio (95% CI)
Afgh1/a*	NA	NA	NA
Afgh1/b*	NA	NA	NA
Afgh1/c*	NA	NA	NA
Afgh2/a	0<br>0/607	98.8<br>724/733	∞†
Afgh2/b	0<br>0/594	100<br>466/466	∞†
Cam1/a‡	78.3<br>313/400<br>–<br>–	(1): 70.7<br>494/699<br>(2): 79.5<br>617/776	(1): 0.67<br>(0.17, 2.66)<br>(2): 1.08<br>(0.28, 4.1)
Cam1/b‡	80.4<br>226/281<br>–<br>–	(1): 81.0<br>757/933<br>(2): 77.8<br>692/889	(1): 1.04<br>(0.23, 4.7)<br>(2): 0.85<br>(0.15, 4.7)
Ghan1/a*	NA	NA	NA
Ghan1/b*	NA	NA	NA
Nige1§	1.7<br>27/1,634	(1): 29.9<br>472/1,576<br>(2): 23.2<br>428/1,844<br>(3): 15.8<br>237/1,502	(1): 25.5<br>(7.5, 86)<br>(2): 18.0<br>(5.0, 64)<br>(3): 11.2<br>(3.08, 40)
Tanz1/a	7.3<br>50/689	48.4<br>363.750	12.0<br>(4.2, 34.6)
Tanz1/b	12.7<br>71/559	43.1<br>167/387	5.2<br>(2.26, 12.0)
Tanz1/c	31.3<br>156/498	71.5<br>409/572	5.5<br>(2.30, 13.2)
Tanz2‖	0<br>0/16,068	(1): 39.1<br>5,556/14,216<br>(2): 39.7<br>6,332/15,931<br>(3): 40.6<br>5,673/13,972	∞†
Uga1	7.3<br>15,285/210,758	52.9<br>117,350/221,755	14.4<br>(6.1, 33.9)
Uga2/a	0<br>0/10,625	96.0<br>1,150/1,198	∞†
Uga2/b	0<br>0/2,444	98.7<br>7,736/7,840	∞†
Uga3	0<br>0/8,109	97.3<br>10,078/10,357	∞†

CI = confidence interval; mRDT = malaria rapid diagnostic test; NA = not applicable.

* Afgh1 and Ghan1 studies individually randomized patients to malaria diagnostic method and are not included in this analysis.

† Odds ratio undefined; approaches infinity.

‡ Cam1/a and Cam1/b each had two interventions: (R1) mRDTs supplied with basic training, and (R2) mRDTs supplied with enhanced training.

§ Nige1 had three interventions: (R1) mRDTs supplied with basic training, (R2) mRDTs supplied with enhanced training, and (R3) mRDTs supplied with enhanced training plus school-based activities.

‖ Tanz2 had three interventions: (R1) mRDTs supplied with basic training, (R2) mRDTs supplied with enhanced training, and (R3) mRDTs supplied with enhanced training plus patient sensitization.

Supplemental Table 2

Patients under age 5 years tested by any malaria diagnostic test

Study (by setting)	Without mRDT interventions<br>Percentage numerator/denominator	With mRDT interventions<br>Percentage numerator/denominator	Odds ratio (95% CI)
Afgh1/a*	NA	NA	NA
Afgh1/b*	NA	NA	NA
Afgh1/c*	NA	NA	NA
Afgh2/a	0.0<br>0/47	100.0<br>76/76	∞†
Afgh2/b	0.0<br>0/20	100.0<br>15/15	∞†
Cam1/a‡	85.2<br>109/128	(1): 64.4<br>116/680<br>(2): 81.2<br>190/234	(1): 0.32<br>(0.04, 2.42)<br>(2): 0.75<br>(0.12, 4.7)
Cam1/b‡	85.2<br>92/108	(1): 84.0<br>352/419<br>(2): 77.4<br>291/376	(1): 0.91<br>(0.18, 4.7)<br>(2): 0.60<br>(0.09, 3.85)
Ghan1/a*	NA	NA	NA
Ghan1/b*	NA	NA	NA
Nige1§	2.0<br>6/299	(1): 32.8<br>38/116<br>(2): 30.9<br>43/139<br>(3): 27.7<br>38/137	(1): 23.8<br>(5.46, 103)<br>(2): 21.9<br>(4.96, 96.5)<br>(3): 18.7<br>(3.27, 107)
Tanz1/a	3.5<br>14/405	50.4<br>259/514	28.4<br>(7.9, 102)
Tanz1/b	10.7<br>33/308	40.5<br>100/247	5.7<br>(2.41, 13.3)
Tanz1/c	32.2<br>103/320	72.2<br>268/371	5.48<br>(2.16, 13.9)
Tanz2‖	0.0<br>0/6,559<br>–<br>–<br>–<br>–	(1): 37.7<br>1,981/5,270<br>(2): 38.5<br>2,346/6,101<br>(3): 39.1<br>1,820/4,649	∞†<br>–<br>–<br>–<br>–<br>–
Uga1	8.9<br>5,774/64,825	61.0<br>40,978/67,159	16.0<br>(6.1, 42)
Uga2/a	0.0<br>0/2,444	96.0<br>1,150/1,198	∞†
Uga2/b	0.0<br>0/10,625	98.7<br>7,736/1,198	∞†
Uga3	0.0<br>0/2,761	98.3<br>3,829/3,895	∞†

CI = confidence interval; mRDT = malaria rapid diagnostic test; NA = not applicable.

* Afgh1 and Ghan1 studies individually randomized patients to malaria diagnostic method and are not included in this analysis.

† Odds ratio undefined; approaches infinity.

‡ Cam1/a and Cam1/b each had two interventions: (R1) mRDTs supplied with basic training, and (R2) mRDTs supplied with enhanced training.

§ Nige1 had three interventions: (R1) mRDTs supplied with basic training, (R2) mRDTs supplied with enhanced training, and (R3) mRDTs supplied with enhanced training plus school-based activities.

‖ Tanz2 had three interventions: (R1) mRDTs supplied with basic training, (R2) mRDTs supplied with enhanced training, and (R3) mRDTs supplied with enhanced training plus patient sensitization.

Supplemental Table 3

Patients ages 5 years and older tested by any malaria diagnostic test

Study (by setting)	Without mRDT interventions Percentage numerator/denominator	With mRDT interventions Percentage numerator/denominator	Odds ratio (95% CI)
Afgh1/a*	NA	NA	NA
Afgh1/b*	NA	NA	NA
Afgh1/c*	NA	NA	NA
Afgh2/a	0.0	98.6	∞†
	0/558	633/642	
Afgh2/b	0.0	100.0	∞†
	0/573	449/449	
Cam1/a‡	75.0	(1): 72.8	(1): 0.89
	204/272	378/519	(0.28, 2.90)
	–	(2): 78.8	(2): 1.24
	–	427/542	(0.37, 4.1)
Cam1/b‡	77.5	(1): 78.6	(1): 1.07
	134/173	403/513	(0.24, 4.7)
	–	(2): 78.2	(2): 1.04
	–	401/513	(0.20, 5.5)
Ghan1/a*	NA	NA	NA
Ghan1/b*	NA	NA	NA
Nige1§	1.5	(1): 29.5	(1): 26.9
	20/1,306	424/1,438	(7.3, 99)
	–	(2): 22.3	(2): 18.5
	–	375/1,679	(4.8, 71)
	–	(3): 14.3	(3): 10.7
	–	191/1,336	(2.85, 40)
Tanz1/a	12.7	44.1	5.4
	36/284	104/236	(1.77, 16.6)
Tanz1/b	15.1	47.9	5.1
	38/251	67/140	(1.92, 13.8)
Tanz1/c	29.8	70.1	5.5
	53/178	141/201	(2.21, 13.9)
Tanz2‖	0.0	(1): 40.0	∞†
	0/9,429	3,572/8,926	–
	–	(2): 40.6	–
	–	3,975/9,787	–
	–	(3): 41.4	–
	–	3,849/9,301	–
Uga1	6.5	49.9	14.4
	9,298/143,834	74,313/148,980	(6.23, 33.3)
Uga2/a	NA	NA	NA
Uga2/b	NA	NA	NA
Uga3	0.0	96.7	∞†
	0/5,625	6,156/6,369	

CI = confidence interval; mRDT = malaria rapid diagnostic test; NA = not applicable.
* Afgh1 and Ghan1 studies individually randomized patients to malaria diagnostic method and are not included in this analysis.
† Odds ratio undefined; approaches infinity.
‡ Cam1/a and Cam1/b each had two interventions: (R1) mRDTs supplied with basic training, and (R2) mRDTs supplied with enhanced training.
§ Nige1 had three interventions: (R1) mRDTs supplied with basic training, (R2) mRDTs supplied with enhanced training, and (R3) mRDTs supplied with enhanced training plus school-based activities.
‖ Tanz2 had three interventions: (R1) mRDTs supplied with basic training, (R2) mRDTs supplied with enhanced training, and (R3) mRDTs supplied with enhanced training plus patient sensitization.

Supplemental Table 4

Patients prescribed ACT

Study (by setting)	Without mRDT interventions<br>Percentage numerator/denominator	With mRDT interventions<br>Percentage numerator/denominator	Odds ratio (95% CI)
Afgh1/a	42.0	36.1	0.78
	843/2,005	740/2,048	(0.68, 0.90)
Afgh1/b	24.4	20.1	0.78
	126/517	106/527	(0.54, 1.13)
Afgh1/c	87.6	34.7	0.07
	283/323	114/329	(0.02, 0.25)
Afgh2/a	77.9	26.6	0.10
	471/605	188/708	(0.04, 0.25)
Afgh2/b	95.9	0.6	0.00
	567/591	3/463	(0.00, 0.00)
Cam1/a*	79.5	(1): 43.5	(1): 0.20
	314/395	294/676	(0.06, 0.61)
	–	(2): 37.9	(2): 0.16
	–	286/754	(0.05, 0.52)
Cam1/b*	69.8	(1): 55.1	(1): 0.53
	194/278	496/900	(0.28, 1.02)
	–	(2): 46.5	(2): 0.38
	–	408/877	(0.17, 0.84)
Ghan1/a	63.7	62.3	0.94
	1,210/1,900	1,180/1,893	(0.83, 1.08)
Ghan1/b	91.6	69.0	0.20
	1,577/1,722	1,188/1,721	(0.07, 0.62)
Nige1†	56.9	(1): 49.3	(1): 0.74
	934/1,642	717/1,453	(0.40, 1.35)
	–	(2): 45.4	(2): 0.63
	–	828/1,823	(0.38, 1.03)
	–	(3): 54.2	(3): 0.90
	–	808/1,409	(0.51, 1.57)
Tanz1/a‡	20.1	8.5	0.37
	138/688	64/750	(0.17, 0.79)
Tanz1/b‡	49.2	25.5	0.35
	275/559	99/388	(0.20, 0.61)
Tanz1/c‡	57.0	35.1	0.41
	284/498	201/572	(0.24, 0.68)
Tanz2§	43.6	(1): 15.8	(1): 0.24
	7,008/16,068	2,246/14,217	(0.15, 0.40)
	–	(2): 11.6	(2): 0.17
	–	1,846/15,931	(0.10, 0.28)
	–	(3): 9.5	(3): 0.14
	–	1,329/13,973	(0.08, 0.22)
Uga1	53.8	50.8	0.89
	113,411/210,758	112,673/221,755	(0.67, 1.19)
Uga2/a	95.1	8.2	0.00
	2,310/2,428	94/1,149	(0.00, 0.01)
Uga2/b	99.2	45.7	0.01
	10,500/10,589	3,512/7,677	(0.00, 0.01)
Uga3	99.1	60.0	0.01
	7,981/8,055	5,933/9,987	(0.01, 0.04)

CI = confidence interval; mRDT = malaria rapid diagnostic test; NA = not applicable. Table presents the percentage of patients prescribed artemisinin-based combination therapy (ACT) except for: Afgh1 and Afgh2, where the percentage prescribed all antimalarials is presented to account for *Plasmodium vivax* treatment; and Nige1 without mRDT interventions only, where ACT or sulfadoxine-pyrimethamine (SP) is presented to reflect treatment practices at the time of data collection.

* Cam1/a and Cam1/b each had two interventions: (R1) mRDTs supplied with basic training, and (R2) mRDTs supplied with enhanced training.

† Nige1 had three interventions: (R1) mRDTs supplied with basic training, (R2) mRDTs supplied with enhanced training, and (R3) mRDTs supplied with enhanced training plus school-based activities.

‡ ACTs obtained by patient at the consultation (ACTs prescribed not available).

§ Tanz2 had three interventions: (R1) mRDTs supplied with basic training, (R2) mRDTs supplied with enhanced training, and (R3) mRDTs supplied with enhanced training plus patient sensitization.

Supplemental Table 5

Patients in scenarios with mRDT interventions prescribed ACT, by malaria test result and not tested

Study (by setting)	Percentage patients prescribed an ACT (95% CI) numerator/denominator		
	Positive test result	Negative test result	Not tested
Afgh1/a*†‡	99.5 (97.9, 99.9)<br>552/555	12.6 (5.1, 27.7)<br>188/1,493	NA
Afgh1/b*†‡	–	19.2 (1.7, 76.0)<br>100/521	NA
Afgh1/c*†‡	–	34.5 (8.4, 75.1)<br>113/328	NA
Afgh2/a*‡	82.7 (63.3, 93.0)<br>163/197	4.0 (1.7, 9.2)<br>20/503	–
Afgh2/b*‡	–	0.4 (0.1, 1.3)<br>2/459	–
Cam1/a§	(1): 70.2 (37.3, 90.3)<br>33/47	(1): 39.2 (13.4, 72.8)<br>167/426	(1): 45.2 (28.7, 62.7)<br>89/197
	(2): 79.6 (62.1, 90.3)<br>117/147	(2): 24.6 (7.4, 57.2)<br>110/447	(2): 35.3 (6.1, 82.0)<br>54/153
Cam1/b§	(1): 72.4 (58.2, 83.1)<br>254/351	(1): 42.5 (29.3, 56.9)<br>157/369	(1): 48.5 (40.5, 56.6)<br>81/167
	(2): 70.1 (54.6, 82.0)<br>246/351	(2): 18.3 (8.8, 34.2)<br>57/312	(2): 51.6 (28.6, 73.9)<br>99/192
Ghan1/a†	98.2 (96.7, 99.0)<br>584/595	45.9 (43.2, 48.6)<br>596/1,298	(1): 45.0 (29.1, 61.9)<br>453/1,007
	–<br>–	–<br>–	(2): 48.2 (38.3, 58.2)<br>674/1,399
	–<br>–	–<br>–	(3): 56.9 (46.1, 67.1)<br>710/1,248
Ghan1/b†	97.5 (88.0, 99.5)<br>692/710	49.1 (1.0, 98.9)<br>496/1,011	NA
Nige1‖	(1): 74.4 (68.3, 79.6)<br>238/320	(1): 21.4 (10.3, 39.3)<br>24/112	NA
	(2): 66.9 (48.4, 81.3)<br>95/142	(2): 21.6 (10.8, 38.5)<br>59/273	
	(3): 60.2 (40.3, 77.2)<br>59/98	(3): 26.5 (11.3, 50.4)<br>36/136	
Tanz1/a¶	18.2 (7.0, 39.7)<br>14/77	4.9 (2.5, 9.6)<br>14/283	9.3 (4.9, 17.1)<br>36/387
Tanz1/b¶	94.4 (56.6, 99.5)<br>17/18	13.4 (8.1, 21.4)<br>20/149	28.2 (18.3, 40.8)<br>62/220
Tanz1/c¶	68.2 (53.4, 80.1)<br>131/192	5.1 (1.9, 12.9)<br>11/217	36.2 (17.3, 60.7)<br>59/163
Tanz2#	(1): 78.8 (64.6, 88.3)<br>1,146/1,455	(1): 17.0 (10.7, 26.0)<br>685/4,024	(1): 4.6 (2.2, 9.6)<br>401/8,660
	(2): 80.2 (65.2, 89.8)<br>1,361/1,696	(2): 4.2 (1.8, 9.7)<br>191/4,541	(2): 3.0 (1.2, 7.0)<br>286/9,599
	(3): 75.6 (50.3, 90.4)<br>944/1,249	(3): 3.6 (1.6, 8.2)<br>157/4,332	(3): 2.6 (1.2, 5.7)<br>218/8,299
Uga1	93.2 (89.7, 95.5)<br>75,808/81,359	2.7 (2.1, 3.5)<br>977/35,711	34.2 (23.1, 47.3)<br>35,717/104,405
Uga2/a	66.7 (41.7, 84.8)<br>24/36	3.1 (1.4, 6.4)<br>32/1,041	69.6 (48.7, 84.6)<br>32/46
Uga2/b	98.0 (96.9, 98.7)<br>3,318/3,385	1.1 (0.6, 1.8)<br>43/4,067	93.1 (80.4, 97.8)<br>95/102
Uga3	97.9 (96.5, 98.7)<br>5,551/5,670	1.2 (0.8, 1.9)<br>47/3,868	96.4 (91.4, 98.5)<br>269/279

ACT = artemisinin-based combination therapy; CI = confidence interval; mRDT = malaria rapid diagnostic tests; NA = not applicable.

* Table presents the percentage of patients in scenarios with mRDT interventions prescribed ACT except for Afgh1 and Afgh2, where the percentage prescribed all antimalarials is presented to account for *Plasmodium vivax* treatment.

† Patients in the Afgh1 and Ghan1 studies were individually randomized to malaria diagnostic method; data are not included in this analysis because all patients in scenarios with mRDT interventions were tested.

‡ Data not included in the analysis when denominators were less than 10.

§ Cam1/a and Cam1/b each had two interventions: (R1) mRDTs supplied with basic training, and (R2) mRDTs supplied with enhanced training.

‖ Nige1 had three interventions: (R1) mRDTs supplied with basic training, (R2) mRDTs supplied with enhanced training, and (R3) mRDTs supplied with enhanced training plus school-based activities.

¶ ACTs obtained by patient at the consultation; data on ACTs prescribed not available in Tanz1.

# Tanz2 had three interventions: (R1) mRDTs supplied with basic training, (R2) mRDTs supplied with enhanced training, and (R3) mRDTs supplied with enhanced training plus patient sensitization.

Supplemental Table 6

Patients prescribed a systemic antibacterial

	Without mRDT interventions	With mRDT interventions	
Study (by setting)	Percentage numerator/denominator	Percentage numerator/denominator	Odds ratio (95% CI)
Afgh1/a	38.1	40.8	1.12
	763/2,005	836/2,048	(0.97, 1.03)
Afgh1/b	73.3	78.9	1.36
	379/517	416/527	(0.76, 2.43)
Afgh1/c	14.2	57.1	8.0
	46/323	188/329	(2.70, 23.9)
Afgh2/a	18.2	54.1	5.3
	110/605	383/708	(2.28, 12.4)
Afgh2/b	48.4	68.5	2.32
	286/591	317/463	(0.85, 6.3)
Cam1/a*	72.8	(1): 78.5	(1): 1.36
	287/394	534/680	(0.68, 2.84)
	–	(2): 78.5	(2): 1.36
	–	595/757	(0.76, 2.43)
Cam1/b*	50.4	(1): 56.9	(1): 1.30
	140/278	510/897	(0.48, 3.53)
	–	(2): 47.3	(2): 0.89
	–	415/877	(0.35, 2.27)
Ghan1/a	30.8	32.1	1.06
	586/1,900	608/1,896	(0.92, 1.21)
Ghan1/b	28.0	32.6	1.24
	483/1,723	560/1,719	(0.90, 1.72)
Nige1†	23.2	(1): 13.6	(1): 0.52
	382/1,642	199/1,467	(0.29, 0.93)
	–	(2): 18.2	(2): 0.73
	–	330/1,817	(0.46, 1.17)
	–	(3): 15.1	(3): 0.59
	–	223/1,474	(0.34, 1.03)
Tanz1/a‡	29.7	44.7	1.92
	204/688	335/749	(1.20, 3.08)
Tanz1/b‡	35.2	56.4	2.38
	197/688	219/388	(1.54, 3.68)
Tanz1/c‡	33.1	49.0	1.94
	165/498	280/572	(1.32, 3.83)
Tanz24§	61.5	(1): 73.2	(1): 1.71
	9,875/16,068	10,407/14,217	(1.25, 2.35)
	–	(2): 75.4	(2): 1.92
	–	12,014/15,931	(1.42, 2.61)
	–	(3): 70.5	(3): 1.50
	–	9,853/13,973	(1.19, 1.89)
Uga1	53.7	57.9	1.18
	113,102/210,758	128,406/221,755	(0.79, 1.77)
Uga2/a||	NA	NA	NA
Uga2/b||	NA	NA	NA
Uga3	19.4	34.9	2.24
	48/248	87/249	(1.41, 3.54)

CI = confidence interval; mRDT = malaria rapid diagnostic test; NA = not applicable.

* Cam1/a and Cam1/b each had two interventions: (R1) mRDTs supplied with basic training, and (R2) mRDTs supplied with enhanced training.

† Nige1 had three interventions: (R1) mRDTs supplied with basic training, (R2) mRDTs supplied with enhanced training, and (R3) mRDTs supplied with enhanced training plus school-based activities.

‡ Any antibacterials obtained by patients at the consultation; data on systemic antibacterials prescribed not available in Tanz1.

§ Tanz2 had three interventions: (R1) mRDTs supplied with basic training, (R2) mRDTs supplied with enhanced training, and (R3) mRDTs supplied with enhanced training plus patient sensitization.

|| Community health workers in Uga2 were not permitted to prescribe antibacterials medicines, so this study is not included in this analysis.

Supplemental Table 7

Patients in scenarios with mRDT interventions prescribed a systemic antibacterial, by malaria test result and not tested

	Percentage prescribed an antibacterial (95% CI) numerator/denominator		
Study (by setting)	Positive test result	Negative test result	Not tested
Aghg1/a*	3.6 (1.8, 7.1)<br>20/555	54.7 (37.9, 70.4)<br>816/1,493	NA
Afgh1/b*†	–	70.7 (60.0, 91.1)<br>415/521	NA
Afgh1/c*†	–	57.3 (23.0, 85.8)<br>188/328	NA
Afgh2/a†	8.1 (4.8, 13.5)<br>16/197	72.8 (49.9, 87.8)<br>366/503	–
Afgh2/b†	–	68.8 (41.9, 87.1)<br>316/459	–
Cam1/a‡	(1): 66.0 (48.2, 80.1)<br>31/47<br>(2): 74.7 (65.3, 82.2)<br>109/146	(1): 79.9 (65.9, 89.1)<br>341/427<br>(2): 83.1 (75.4, 88.9)<br>375/451	(1): 80.0 (57.5, 92.2)<br>160/200<br>(2): 69.3 (64.6, 73.6)<br>106/153
Cam1/b‡	(1): 58.0 (42.4, 72.2)<br>203/350<br>(2): 43.7 (31.7, 56.5)<br>153/350	(1): 63.5 (46.9, 77.4)<br>(2): 52.6 (45.1, 60.0)<br>164/312<br>–	(1): 42.5 (25.2, 61.9)<br>71/167<br>(2): 47.9 (28.2, 68.3)<br>92/192
Ghan1/a*	14.6 (12.0, 17.7)<br>87/595	40.0 (37.4, 42.7)<br>521/1,301	NA
Ghan1/b*	18.0 (11.1, 27.9)<br>128/710	42.8 (12.4, 79.9)<br>432/1,009	NA
Nige1§	(1): 17.5 (13.3, 22.7)<br>55/314<br>(2): 14.9 (8.5, 24.7)<br>21/141<br>(3): 20.2 (8.5, 41.0)<br>20/99	(1): 26.1 (12.2, 47.4)<br>29/111<br>(2): 13.3 (6.7, 24.6)<br>37/279<br>(3): 21.6 (13.9, 32.2)<br>29/134	(1): 11.1 (5.6, 20.9)<br>114/1,026<br>(2): 19.5 (14.9, 25.2)<br>271/1,388<br>(3): 13.9 (8.9, 21.3)<br>172/1,233
Tanz1/a‖	31.6 (17.3, 50.5)<br>24/76	53.0 (42.2, 63.6)<br>150/283	41.3 (30.4, 52.2)<br>160/387
Tanz1/b‖	44.4 (11.4, 83.3)<br>8/18	58.4 (47.2, 68.8)<br>87/149	56.4 (44.7, 67.4)<br>124/220
Tanz1/c‖	30.7 (20.8, 42.9)<br>59/192	61.8 (50.4, 71.9)<br>134/217	53.4 (39.4, 66.8)<br>87/163
Tanz2¶	(1): 26.5 (13.4, 45.8)<br>386/1,455<br>(2): 36.1 (25.4, 48.3)<br>612/1,696<br>(3): 26.3 (18.0, 36.6)<br>328/1,249	(1): 74.2 (67.1, 80.2)<br>2,985/4,024<br>(2): 77.7 (69.9, 83.9)<br>3,527/4,541<br>(3): 74.7 (69.2, 79.6)<br>3,238/4,332	(1): 80.6 (75.5, 84.8)<br>6,979/6,880<br>(2): 81.3 (75.1, 86.2)<br>7,800/9,599<br>(3): 74.9 (68.6, 80.3)<br>6,214/8,299
Uga1	40.5 (30.6, 51.2)<br>32,935/81,359	69.3 (65.3, 73,0)<br>24,737/35,711	66.3 (60.5, 71.7)<br>69,178/104,405
Uga2/a#	NA	NA	NA
Uga2/b#	NA	NA	NA
Uga3†	23.6 (16.3, 33.0)<br>30/127	46.0 (37.7, 54.6)<br>52/113	–

CI = confidence interval; mRDT = malaria rapid diagnostic test; NA = not applicable.

* Patients in the Afgh1 and Ghan1 studies were individually randomized to malaria diagnostic method; data are not included in this analysis because all patients in scenarios with mRDT interventions were tested.

† Data not included in the analysis when denominators were less than 10.

‡ Cam1/a and Cam1/b each had two interventions: (R1) mRDTs supplied with basic training, and (R2) mRDTs supplied with enhanced training.

§ Nige1 had three interventions: (R1) mRDTs supplied with basic training, (R2) mRDTs supplied with enhanced training, and (R3) mRDTs supplied with enhanced training plus school-based activities.

‖ Any antibacterials obtained by patients at the consultation; data on systemic antibacterials prescribed not available in Tanz1.

¶ Tanz2 had three interventions: (R1) mRDTs supplied with basic training, (R2) mRDTs supplied with enhanced training, and (R3) mRDTs supplied with enhanced training plus patient sensitization.

# Community health workers in Uga2 were not permitted to prescribe antibacterials medicines, so this study is not included in this analysis.

Supplemental Table 8

Patients prescribed an antimalarial and an antibacterial

Study (by setting)	Without mRDT interventions<br>Percentage numerator/denominator	With mRDT interventions<br>Percentage numerator/denominator	Odds ratio (95% CI)
Afgh1/a	1.2<br>25/2,005	1.5<br>30/2,048	1.18 (0.66, 2.11)
Afgh1/b	10.6<br>55/517	8.7<br>46/527	0.80 (0.54, 1.19)
Afgh1/c	4.6<br>15/323	4.3<br>14/329	0.91 (0.50, 1.68)
Afgh2/a	47.4<br>280/51	0.2<br>1/463	0.00 (0.00, 0.02)
Afgh2/b	16.7<br>101/605	3.1<br>22/708	0.16 (0.06, 0.40)
Cam1/a*	66.1<br>259/392	(1): 37.7<br>253/672	(1): 0.31 (0.15, 0.65)
	–<br>–	(2): 32.8<br>247/753	(2): 0.25 (0.10, 0.61)
Cam2/b*	43.9<br>122/278	(1): 40.1<br>359/896	(1): 0.85 (0.33, 2.24)
	–<br>–	(2): 28.1<br>247/875	(2): 0.50 (0.19, 1.31)
Ghan1/a	12.3<br>234/1,899	12.4<br>235/1,893	1.01 (0.83, 1.22)
Ghan1/b	23.8<br>410/1,722	15.3<br>263/1,719	0.58 (0.32, 1.04)
Nige1†	14.7<br>241/1,642	(1): 12.3<br>174/1,459	(1): 0.81 (0.48, 1.39)
	–<br>–	(2): 14.0<br>253/1,805	(2): 0.95 (0.58, 1.55)
	–<br>–	(3): 11.9<br>175/1,472	(3): 0.78 (0.51, 1.21)
Tanz1/a‡	6.8<br>47/688	4.1<br>31/749	0.59 (0.27, 1.28)
Tanz1/b‡	18.2<br>102/559	12.9<br>50/388	0.66 (0.36, 1.22)
Tanz1/c‡	15.5<br>77/498	14.7<br>84/572	0.94 (0.49, 1.82)
Tanz2§	24.2<br>3,891/16,068	(1): 6.9<br>974/14,217	(1): 0.23 (0.14, 0.37)
	–<br>–	(2): 5.7<br>908/15,931	(2): 0.19 (0.12, 0.31)
	–<br>–	(3): 3.6<br>500/13,973	(3): 0.12 (0.07, 0.19)
Uga1	23.8<br>50,098/210,758	23.5<br>52,212/221,755	0.99 (0.59, 1.65)
Uga2/a‖	NA	NA	NA
Uga2/b‖	NA	NA	NA
Uga3	19.4<br>48/248	14.2<br>33/233	0.69 (0.45, 1.06)

CI = confidence interval; mRDT = malaria rapid diagnostic test; NA = not applicable.

* Cam1/a and Cam1/b each had two interventions: (R1) mRDTs supplied with basic training, and (R2) mRDTs supplied with enhanced training.

† Nige1 had three interventions: (R1) mRDTs supplied with basic training, (R2) mRDTs supplied with enhanced training, and (R3) mRDTs supplied with enhanced training plus school-based activities.

‡ Antimalarials and any antibacterials obtained by patients at the consultation; data on medicines prescribed not available in Tanz1.

§ Tanz2 had three interventions: (R1) mRDTs supplied with basic training, (R2) mRDTs supplied with enhanced training, and (R3) mRDTs supplied with enhanced training plus patient sensitization.

‖ Community health workers in Uga2 were not permitted to prescribe antibacterials medicines, so this study is not included in this analysis.

Supplemental Table 9

Patients prescribed an antimalarial or an antibacterial

	Without mRDT interventions	With mRDT interventions	
Study (by setting)	Percentage numerator/denominator	Percentage numerator/denominator	Odds ratio (95% CI)
Afgh1/a	78.9	75.5	0.83 (0.74, 0.93)
	1,581/2,005	1,546/2,048	
Afgh1/b	87.0	90.3	1.39 (0.73, 2.66)
	450/517	476/527	
Afgh1/c	97.2	87.5	0.20
	314/323	288/329	0.08, 0.48)
Afgh2/a	97.0	68.9	0.07 (0.03, 0.19)
	573/591	319/463	
Afgh2/b	79.3	77.5	0.90 (0.32, 2.56)
	480/605	549/708	
Cam1/a*	94.2	(1): 92.5	(1): 0.73 (0.19, 2.76)
	372/394	629/680	
	–	(2): 90.9	(2): 0.59 (0.16, 2.14)
	–	687/757	
Cam1/b*	97.1	(1): 88.1	(1): 0.22 (0.10, 0.49)
	270/278	790/897	
	–	(2): 79.8	(2): 0.12 (0.05, 0.27)
	–	700/877	
Ghan1/a	82.9	82.2	0.95 (0.80, 1.12)
	1,575/1,899	1,557/1,894	
Ghan1/b	97.0	85.7	0.21 (0.10, 0.48)
	1,670/1,722	1,501/1,719	
Nige1†	84.5	(1): 81.7	(1): 0.82 (0.37, 1.79)
	1,369/1,642	1158/1,460	
	–	(2): 80.4	(2): 0.75 (0.41, 1.37)
	–	1,451/1,805	
	–	(3): 86.8	(3): 1.20 (0.49, 2.93)
	–	1,278/1,473	
Tanz1/a‡	46.2	52.7	1.30 (0.81, 2.09)
	318/688	395/749	
Tanz1/b‡	68.7	70.4	1.08 (0.61, 1.93)
	384/559	273/388	
Tanz1/c‡	79.1	72.2	0.69 (0.40, 1.16)
	394/498	413/572	
Tanz2§	87.9	(1): 85.4	(1): 0.81 (0.61, 1.08)
	14,125/16,068	12,146/14,217	
	–	(2): 84.4	(2): 0.74 (0.55, 1.01)
	–	13,446/15,931	
	–	(3): 79.0	(3): 0.52 (0.40, 0.66)
	–	11,032/13,973	
Uga1	85.5	87.0	1.13 (0.67, 1.91)
	180,106/210,758	192,831/221,755	
Uga2/a‖	NA	NA	NA
Uga2/b‖	NA	NA	NA
Uga3	100.0	78.7	0.00¶
	248/248	188/239	

CI = confidence interval; mRDT = malaria rapid diagnostic test; NA = not applicable.

* Cam1/a and Cam1/b each had two interventions: (R1) mRDTs supplied with basic training, and (R2) mRDTs supplied with enhanced training.

† Nige1 had three interventions: (R1) mRDTs supplied with basic training, (R2) mRDTs supplied with enhanced training, and (R3) mRDTs supplied with enhanced training plus school-based activities.

‡ Antimalarials and any antibacterials obtained by patients at the consultation; data on medicines prescribed not available in Tanz1.

§ Tanz2 had three interventions: (R1) mRDTs supplied with basic training, (R2) mRDTs supplied with enhanced training, and (R3) mRDTs supplied with enhanced training plus patient sensitization.

‖ Community health workers in Uga2 were not permitted to prescribe antibacterials medicines, so this study is not included in this analysis.

¶ Odds ratio undefined; approaches zero.

Supplemental Table 10

Patients prescribed a systemic antihelminthic, and patients prescribed a systemic antifungal

	Antihelminthics		Antifungals	
	Without mRDT interventions	With mRDT interventions	Without mRDT interventions	With mRDT interventions
Study (by setting)	Percentage numerator/denominator	Percentage numerator/denominator	Percentage numerator/denominator	Percentage numerator/denominator
Afgh1/a	0.5 (10/2,005)	0.3 (7/2,048)	0 (0/2,005)	0 (0/2,048)
Afgh1/b	0.8 (4/517)	0.8 (4/527)	0 (0/517)	0 (0/527)
Afgh1/c	0.0 (0/323)	0.3 (1/329)	0 (0/323)	0 (0/329)
Afgh2/a*	NA	NA	NA	NA
Afgh2/b*	NA	NA	NA	NA
Cam1/a†	11.4 (45/394)<br>–<br>–	(1): 4.3 (29/680)<br>(2): 8.5 (76/897)	0.8 (3/394)<br>–<br>–	(1): 1.2 (8/680)<br>(2): 1.9 (17/897)
Cam1/b†	8.3 (23/278)	(1): 7.1 (54/757)<br>(2): 10.4 (91/877)	1.4(4/278)	(1): 0.4 (3/757)<br>(2): 2.6 (23/877)
Ghan1/a	31.6 (600/1,900)	33.3 (631/1,896)	0.6 (11/1,900)	0.7 (13/1,896)
Ghan1/b	8.6 (149/1,723)	11.3 (195/1,719)	0.1 (1/1,723)	0.3 (6/1,719)
Nige1‡	2.0 (33/1,642)	(1): 0.3 (4/1,588)<br>(2): 0.3 (5/1,850)<br>(3): 1.3 (19/1,508)	0.1 (1/1642)	(1): 0 (0/1,588)<br>(2): 0 (0/1,850)<br>(3): 0 (0/1,508)
Tanz1/a§	1.7 (12/688)	4.5 (34/749)	0 (0/688)	0 (0/749)
Tanz1/b§	3.2 (18/559)	7.2 (28/388)	0 (0/559)	0 (0/388)
Tanz1/c§	2.2 (11/498)	3.1 (18/572)	0 (0/498)	0 (0/572)
Tanz2*	NA	(1) NA<br>(2) NA<br>(4) NA	NA	(1) NA<br>(3) NA<br>(2) NA
Uga1	14.6 (30,865/210,758)	16.1 (35,683/221,755)	0.1 (195/210,758)	0.1 (318/221,755)
Uga2/a*	NA	NA	NA	NA
Uga2/b*	NA	NA	NA	NA
Uga3	4.0 (10/248)	5.2 (13/249)	0 (0/248)	0 (0/249)

mRDT = malaria rapid diagnostic test; NA = not applicable.

* Data on systemic antihelminthics and antifungals prescribed were not collected from Afgh2, Tanz2, and Uga2.

† Cam1/a and Cam1/b each had two interventions: (R1) mRDTs supplied with basic training, and (R2) mRDTs supplied with enhanced training.

‡ Nige1 had three interventions: (R1) mRDTs supplied with basic training, (R2) mRDTs supplied with enhanced training, and (R3) mRDTs supplied with enhanced training plus school-based activities.

§ Systemic antihelminthics and antifungals obtained by patients at the consultation; data on medicines prescribed not available in Tanz1.

SUPPLEMENTAL TABLE 11

Patients prescribed an antipyretic without an antimalarial or an antibiotic

Study (by setting)	Without mRDT interventions<br>Percentage numerator/denominator	With mRDT interventions<br>Percentage numerator/denominator	Odds ratio (95% CI)
Afgh1/a	19.7<br>395/2,005	22.7<br>464/2,048	1.19 (1.07, 1.34)
Afgh1/b	10.8<br>56/517	8.2<br>43/527	0.73 (0.35, 1.53)
Afgh1/c	2.8<br>9/323	12.2<br>40/329	4.8 (2.16, 10.8)
Afgh2/a	1.2<br>7/591	17.3<br>80/463	17.4 (3.41, 89)
Afgh2/b	0.3<br>2/605	11.6<br>82/708	39.5 (12.9, 121)
Cam1/a*	0.5<br>2/392	(1): 4.2<br>28/671	(1): 8.5 (1.34, 54)
	–<br>–	(2): 7.1<br>53/749	(2): 14.9 (2.44, 90)
Cam1/b*	1.8<br>5/278	(1): 3.7<br>33/895	(1): 2.09 (0.66. 6.7)
	–<br>–	(2): 5.8<br>51/874	(2): 3.38 (1.01, 11.4)
Ghan1/a	12.6<br>239/1,899	14.3<br>271/1,893	1.16 (0.96, 1.40)
Ghan1/b	2.4<br>42/1,722	10.2<br>176/1,719	4.6 (1.98, 10.5)
Nige1†	7.6<br>138/1,642	(1): 3.1<br>44/1,459	(1): 0.39 (0.17, 0.89)
	–<br>–	(2): 8.9<br>161/1,825	(2): 1.19 (0.66, 2.13)
	–<br>–	(3): 3.9<br>58/1,489	(3): 0.50 (0.18, 1.39)
Tanz1/a‡	23.7<br>163/688	18.8<br>141/749	0.75 (0.46, 1.23)
Tanz1/b‡	12.7<br>71/559	12.1<br>47/388	0.95 (0.49, 1.83)
Tanz1/c‡	9.4<br>47/498	18.2<br>104/572	2.13 (1.25, 3.64)
Tanz2§	6.4<br>(1,020/15,953)	(1): 10.9<br>1,545/14,217	(1): 1.78 (1.32, 2.41)
	–<br>–	(2): 11.4<br>1,818/15,931	(2): 1.89 (1.34, 2.65)
	–<br>–	(3): 14.1<br>1,964/13,973	(3): 2.39 (1.89, 3.04)
Uga1	10.1<br>21,803/210,758	8.4<br>18,630/221,755	0.79 (0.43, 1.45)
Uga2/a‖	NA	NA	NA
Uga2/b‖	NA	NA	NA
Uga3	0.0<br>0/248	14.6<br>34/233	∞¶

CI = confidence interval; mRDT = malaria rapid diagnostic test; NA = not applicable.

* Cam1/a and Cam1/b each had two interventions: (R1) mRDTs supplied with basic training, and (R2) mRDTs supplied with enhanced training.

† Nige1 had three interventions: (R1) mRDTs supplied with basic training, (R2) mRDTs supplied with enhanced training, and (R3) mRDTs supplied with enhanced training plus school-based activities.

‡ Antipyretics obtained by patients at the consultation; data on medicines prescribed not available in Tanz1.

§ Tanz2 had three interventions: (R1) mRDTs supplied with basic training, (R2) mRDTs supplied with enhanced training, and (R3) mRDTs supplied with enhanced training plus patient sensitization.

‖ Community health workers in Uga2 were not permitted to prescribe antibacterials medicines, so this study is not included in this analysis.

¶ Odds ratio undefined; approaches infinity.

Supplemental Table 12

Patients prescribed three or more medicines

	Without mRDT interventions	With mRDT interventions	
Study (by setting)	Percentage numerator/denominator	Percentage numerator/denominator	Odds ratio (95% CI)
Afgh1/a	16.1<br>322/2,005	15.1<br>309/2,048	0.93 (0.80, 1.08)
Afgh1/b	36.0<br>186/517	34.7<br>183/527	0.95 (0.85, 1.06)
Afgh1/c	60.1<br>194/323	38.3<br>126/329	0.41 (0.19, 0.92)
Afgh2/a	62.3<br>368/591	3.2<br>15/463	0.02 (0.01, 0.05)
Afgh2/b	28.1<br>170/605	10.2<br>72/708	0.29 (0.10, 0.88)
Cam1/a*	87.5<br>350/400	(1): 82.3<br>575/699	(1): 0.66 (0.28, 1.58)
	–<br>–	(2): 79.8<br>621/778	(2): 0.57 (0.26, 1.22)
Cam1/b*	78.6<br>221/281	(1): 71.5<br>667/933	(1): 0.68 (0.33, 1.41)
	–<br>–	(2): 61.3<br>546/891	(2): 0.43 (0.19, 0.98)
Ghan1/a	91.8<br>(1,751/1,907)	91.4<br>(1,740/1,904)	0.95 (0.75, 1.19)
Ghan1/b	90.3 (1,559/1,727)	88.2 (1,521/1,725)	0.80 (0.61, 1.07)
Nige1†	60.8<br>965/1,587	(1): 56.4<br>896/1,588	(1): 0.83 (0.45, 1.54)
	–<br>–	(2): 53.2<br>985/1,850	(2): 0.73 (0.39, 1.38)
	–<br>–	(3): 57.1<br>861/1,508	(3): 0.86 (0.51, 1.44)
Tanz1/a‡	13.8<br>95/688	18.6<br>139/748	1.42 (0.80, 2.53)
Tanz1/b‡	24.2<br>143/498	30.7<br>119/388	1.39 (0.90, 2.14)
Tanz1/c‡	28.7<br>143/498	26.6<br>152/571	0.90 (0.52, 1.57)
Tanz2§	NA	(1): NA<br>(2): NA<br>(3): NA	(1): NA<br>(2): NA<br>(3): NA
Uga1	50.9<br>107,348/210,758	52.2<br>115,850/221,755	1.05 (0.66, 1.68)
Uga2/a‖	NA	NA	NA
Uga2/b‖	NA	NA	NA
Uga3	54.0<br>134/248	60.2<br>150/249	1.29 (0.80, 2.07)

CI = confidence interval; mRDT = malaria rapid diagnostic test; NA = not applicable.

* Cam1/a and Cam1/b each had two interventions: (R1) mRDTs supplied with basic training, and (R2) mRDTs supplied with enhanced training.

† Nige1 had three interventions: (R1) mRDTs supplied with basic training, (R2) mRDTs supplied with enhanced training, and (R3) mRDTs supplied with enhanced training plus school-based activities.

‡ Medicines obtained by patients at the consultation; data on medicines prescribed not available in Tanz1.

§ Tanz2 did not record data on all medications prescribed, so this study is not included in Figure 4D.

‖ Community health workers in Uga2 were not permitted to prescribe antibacterials medicines, so this study is not included in this analysis.

SUPPLEMENTAL TABLE 13

Patients referred to another care provider or health facility

Study (by setting)	Without mRDT interventions Percentage numerator/denominator	With mRDT interventions Percentage numerator/denominator	Odds ratio (95% CI)
Afgh1/a	0.1<br>3/2,005	0.0<br>1/2,048	0.33 (0.03, 3.47)
Afgh1/b	0.0<br>0/323	0.0<br>0/329	–
Afgh1/c	0.2<br>1/517	0.4<br>2/527	1.97 (0.13, 29.1)
Afgh2/a	29.0<br>172/594	64.2<br>299/466	4.4 (2.28, 8.5)
Afgh2/b	23.9<br>145/607	15.7<br>115/732	0.59 (0.19, 1.81)
Cam1*	0.8<br>3/399	(1): 0.9<br>6/695	(1): 1.15 (0.28, 4.7)
	–<br>–	(2): 1.4<br>11/769	(2): 1.92 (0.47, 7.8)
Cam2*	0.4<br>1/280	(1): 1.3<br>12/903	(1): 3.76 (0.70, 20.1)
	–<br>–	(2): 3.5<br>30/864	(2): 10.0 (1.38, 73)
Ghan1/a†	NA	NA	NA
Ghan1/b†	NA	NA	NA
Nige1‡	1.0<br>17/1,630	(1): 1.9<br>30/1,548	(1): 1.88 (0.59, 5.9)
	–<br>–	(2): 1.7<br>31/1,833	(2): 1.63 (0.61, 4.4)
	–<br>–	(3): 3.8<br>57/1,491	(3): 3.77 (0.89, 15.93)
Tanz1/a†	NA	NA	NA
Tanz1/b†	NA	NA	NA
Tanz1/c†	NA	NA	NA
Tanz2†§	NA	(1): NA<br>(2): NA<br>(3): NA	(1): NA<br>(2): NA<br>(3): NA
Uga1†	NA	NA	NA
Uga2/b	1.0<br>109/10,599	35.1<br>821/1,168	52 (34.8, 77)
Uga2/a	13.1<br>314/2,404	70.3<br>2,706/77,16	15.8 (9.3, 27)
Uga3	3.5<br>279/8,039	12.1<br>1,234/10,226	3.82 (0.82, 17.8)

CI = confidence interval; mRDT = malaria rapid diagnostic test; NA = not applicable.

* Cam1/a and Cam1/b each had two interventions: (R1) mRDTs supplied with basic training, and (R2) mRDTs supplied with enhanced training.

† Ghan1, Tanz1, Tanz2, and Uga1 did not record data on referral.

‡ Nige1 had three interventions: (R1) mRDTs supplied with basic training, (R2) mRDTs supplied with enhanced training, and (R3) mRDTs supplied with enhanced training plus school-based activities.

§ Tanz2 had three interventions: (R1) mRDTs supplied with basic training, (R2) mRDTs supplied with enhanced training, and (R3) mRDTs supplied with enhanced training plus patient sensitization.
